# Lenalidomide in Combination with Arsenic Trioxide: an Effective Therapy for Primary Effusion Lymphoma

**DOI:** 10.3390/cancers12092483

**Published:** 2020-09-01

**Authors:** Sara Moodad, Rana El Hajj, Rita Hleihel, Layal Hajjar, Nadim Tawil, Martin Karam, Maguy Hamie, Raghida Abou Merhi, Marwan El Sabban, Hiba El Hajj

**Affiliations:** 1 Department of Internal Medicine, Faculty of Medicine, American University of Beirut, Beirut 202627, Lebanon; shm24@mail.aub.edu (S.M.); rh150@aub.edu.lb (R.H.); mh242@aub.edu.lb (M.H.); 2 Department of Pathology and Laboratory Medicine, Faculty of Medicine, American University of Beirut, Beirut 202627, Lebanon; rh164@aub.edu.lb; 3 Department of Anatomy, Cell Biology, and Physiology, Faculty of Medicine, American University of Beirut, Beirut 202627, Lebanon; lh85@aub.edu.lb (L.H.); me00@aub.edu.lb (M.E.S.); 4 Department of Experimental Pathology, Immunology, and Microbiology, Faculty of Medicine, American University of Beirut, Beirut 202627, Lebanon; nadim.tawil@mail.mcgill.ca (N.T.); martin.karam@mail.mcgill.ca (M.K.); 5 Department of Biology, Faculty of Sciences, GSBT laboratory, Lebanese University, Hadath 31143, Lebanon; raboumerhi@ul.edu.lb

**Keywords:** HHV-8, immunomodulatory drugs, LANA, latent cycle, lytic cycle, lymphoma

## Abstract

Primary effusion lymphoma (PEL) is a rare aggressive subset of non-Hodgkin B cell lymphoma. PEL is secondary to Kaposi sarcoma herpes virus (KSHV) and predominantly develops in serous cavities. Conventional chemotherapy remains the treatment of choice for PEL and yields high response rates with no significant comorbidities. Yet, chemotherapy often fails in achieving or maintaining long-term remission. Lenalidomide (Lena), an immunomodulatory drug, displayed some efficacy in the treatment of PEL. On the other hand, arsenic trioxide (ATO) in combination with other agents effectively treated a number of blood malignancies, including PEL. In this study, we present evidence that the combination of ATO/Lena significantly enhanced survival of PEL mice, decreased the volume of exacerbated ascites in the peritoneum, and reduced tumor infiltration in organs of treated animals. In ex vivo treated PEL cells, ATO/Lena decreased the proliferation and downregulated the expression of KSHV latent viral proteins. This was associated with decreased NF-κB activation, resulting in reactivation of viral replication, downregulation of interleukin-6 (IL-6) and IL-10, inhibition of vascular endothelial growth factor, and apoptosis. Our results elucidate the mechanism of action of ATO/Lena and present it as a promising targeted therapeutic modality in PEL management, which warrants further clinical investigation.

## 1. Introduction

Primary effusion lymphoma (PEL) is an aggressive non-Hodgkin B-Cell lymphoma (NHL) secondary to Kaposi sarcoma herpes virus (KSHV) [1,2]. This virus originally described as acquired immunodeficiency syndrome (AIDS)-associated Kaposi sarcoma [3], was later referred to as human herpesvirus type 8 (HHV-8) [1,2,4]. PEL accounts for around 4% of human immunodeficiency virus (HIV)-associated NHL and less than 1% of non-HIV-related lymphomas [5]. It typically occurs in middle-aged immunocompromised patients, infected either with HIV or recipients of solid-organ transplants, and in elderly patients in HHV-8 endemic areas [6,7,8,9,10]. Clinically, PEL typically presents as body cavity lymphoma with malignant lymphomatous effusions invading peritoneal, pleural, and/or pericardial cavities [2,11]. Extra-cavitary PEL with solid tumor masses is less frequent, and when present, it involves organs adjacent to the cavitary space, regional lymph nodes, and gastrointestinal tract among others [12,13].

KSHV transforms B lymphocytes [5,14] and associates with several malignancies [14,15,16]. In PEL, the KSHV genome is present in all neoplastic cells, and its detection is crucial for definitive diagnosis [15,16,17]. KSHV oncogenesis involves latent and lytic phases [1,17], but the virus mostly resides in B cells in its latent phase, where PEL malignant cells express KSHV-encoded latent proteins including latency-associated nuclear antigen-1 (LANA-1/ORF73), LANA-2/vIRF-3, viral cyclin (v-Cyclin), and viral FLICE inhibitory protein (v-FLIP) [2,17,18,19,20,21]. LANA-1 is crucial for maintaining viral latency and viral episomes [18,19,20,21], and its presence is a standard method for diagnosis of PEL [22,23]. LANA-1 also impairs apoptosis by inhibiting p53 and retinoblastoma (Rb) tumor suppressor proteins, yielding tumor growth and survival [19,24]. It also contributes to the dysregulation of the NOTCH pathway and the inhibition of lytic gene expression [25]. LANA-2, another viral latent protein, impairs p53-mediated apoptosis and prevents p53 SUMOylation leading to cell senescence [26]. It also plays a substantial role in the development of drug resistance to anti-mitotic drugs by reducing polymerized microtubules stability [27]. v-Cyclin, the viral homologue of cellular cyclin D, drives cell cycle progression through binding and constitutively activating cyclin-dependent kinases 4 and 6 (CDK6) resulting in Rb phosphorylation and inactivation [28]. v-FLIP, the viral homologue of FLICE inhibitor protein (c-FLIP) promotes tumor proliferation, cell survival [29], and inhibits apoptosis by blocking death receptor Fas-and TNF mediated caspase activation [30,31]. v-Cyclin and v-FLIP constitutively activate the NF-κB pathway, which plays a key role in PEL tumorigenesis, while maintaining viral latency [32,33,34,35]. In addition to the aforementioned latent viral proteins, PEL cell survival depends on the production of cytokines that serve as autocrine growth factors [36,37]. These include cellular IL-10 and IL-6, which are secreted by PEL cells and promote cellular proliferation and tumor progression [36,37,38].

Given the rarity of PEL, treatment and management of the disease are limited to very few randomized or retrospective studies. An optimal curative regimen for PEL is lacking [11]. Chemotherapy remains the current treatment of PEL patients and leads to good response rates with no significant comorbidities. The chemotherapy regimen includes cyclophosphamide/doxorubicin/vincristine/prednisone (CHOP) or dose adjusted etoposide/prednisolone/vincristine/cyclophosphamide/doxorubicin (DA-EPOCH) [11,39]. In HIV positive PEL patients, highly active antiretroviral therapy (HAART) is often preferred. However, remission is not sustainable due to high relapse rates and resistance of PEL patients to chemotherapy [2,40,41], and the median survival does not exceed six months [11,41].

Lenalidomide (Lena) is an immunomodulatory agent and a thalidomide analogue approved for the treatment of multiple myeloma patients [42,43]. Lena, via its anti-angiogenic, anti-neoplastic, and immune activating properties showed promising efficacy in numerous hematological malignancies [43,44,45]. PEL cells treated in vitro with Lena were cell cycle arrested [46], and immune surface markers downregulated by KSHV were restored as an immune-modulatory mechanism [47]. Among its known mechanisms of action, Lena targeted the IKZF1–IRF4–MYC axis in a cereblon-dependent manner [46]. Lena also degraded IKZF1, a specific B-cell transcription factor, leading to the down-regulation of IRF4 transcript levels and MYC expression, known to be essential for PEL survival and proliferation [46]. A phase I/II clinical study investigating Lena with DA-EPOCH and rituximab is ongoing (NCT02911142). Furthermore, a 77-year-old HIV-negative patient achieved complete remission after receiving Lena. Remission was sustained after 18 months of therapy [48].

Arsenic trioxide (ATO), in combination with various drugs, is a highly effective compound against several hematological malignancies [49,50,51]. Mechanisms of action of ATO are versatile and include induction of apoptosis, inhibition of proliferation and angiogenesis, stimulation of differentiation, and deregulation of cellular redox states [52]. In PEL, ATO combined with interferon-α (IFNα) inhibited cell proliferation and induced apoptosis in vitro [53]. In a PEL murine model, ATO/IFNα prolonged survival, induced apoptosis, and decreased viral latent gene transcripts [54]. Even though results were promising, a cure was not achieved. In this study, we demonstrate that ATO/Lena not only significantly enhanced the survival of PEL mice but also achieved a cure in some mice. In addition, ATO/Lena decreased the volume of effusion in the peritoneum and reduced tumor infiltration in organs of treated animals. In ex vivo treated cells, ATO/Lena decreased proliferation and downregulated the expression of latent viral proteins. This downregulation resulted in decreased NF-κB activation, downregulation of cellular IL-6 and IL-10, reactivation of viral replication, and apoptosis. These results present this combination as an encouraging therapeutic option for PEL patients.

## 2. Results

### 2.1. ATO/Lena Enhances Survival and Reduces Lymphomatous Effusions in PEL Mice

**Figure 1:**
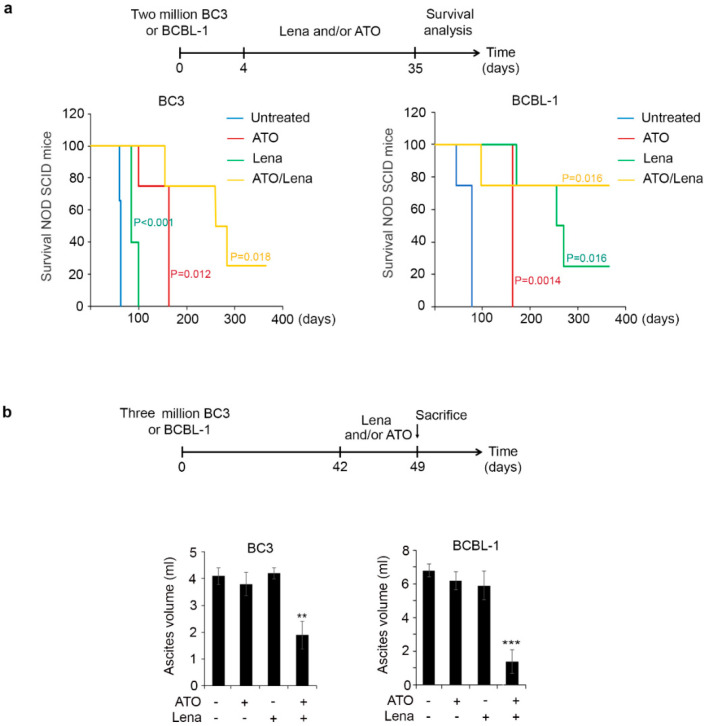
Arsenic trioxide/Lenalidomide (ATO/Lena) enhanced survival and decreased ascites volume in NOD/SCID primary effusion lymphoma (PEL) mice. (**a**) Kaplan-Meier graphs of overall survival curves of BC-3 (left) and BCBL-1 (right) NOD/SCID mice. Mice (*n* = 4 per condition) were injected with 2 million BC-3 or BCBL-1 cells. ATO, Lena, or their combination were administered from day 4 until day 35 post-injection of PEL cells. (**b**) Ascites volume from BC-3 (left) or BCBL-1 (right). PEL mice were allowed to develop ascites for 6 weeks then were treated daily with ATO, Lena, or their combination for one week before sacrifice. (**) indicates *p* < 0.01; and (***) indicates *p* < 0.001.

NOD/SCID mice were intraperitoneally injected with the PEL cells (BC-3 or BCBL-1) (Ethical permit number #15-07-P575). On day 4 post-inoculation of PEL cells, mice were treated with ATO, Lena, or their combination for one month and assessed for survival (timeline described in Figure 1a). In mice injected with BC-3 cells, the median survival significantly increased from 63 days in untreated mice to 163 (*p* = 0.012) in mice treated with ATO or 85 days (*p* < 0.005) in mice treated with Lena alone. The median survival was strikingly increased to 272 days (*p* = 0.018) upon treatment with the ATO/Lena combination, and 25% of treated mice were completely cured, with no effusion formation, after more than one year post-injection of lymphomatous cells. Similarly, in mice injected with BCBL-1 cells, the median survival significantly increased from 78 days in untreated mice to 163 (*p* = 0.014) and 263 days (*p* = 0.016) in mice treated with ATO or Lena single agents, respectively. In Lena treated mice, 25% of mice were cured. Importantly, this median survival reached 360 days in ATO/Lena-treated mice (*p* = 0.016), and 75% of the mice were totally cured after over a year post-injection of malignant BCBL-1 cells. These results demonstrate not only enhanced survival but also a strong curative effect of the ATO/Lena combination.

We then assessed the effect of therapeutic efficacy of ATO/Lena on PEL progression after development of lymphomatous effusion. NOD/SCID mice were thus inoculated with BC-3 or BCBL-1 cells and allowed to develop tumors for six weeks. Mice were then treated with ATO, Lena, or their combination, and the ascites and peritoneal volume were monitored on a daily basis. A moderate and none significant effect on ascites and peritoneal volume was seen in PEL mice injected with BC-3 or BCBL-1 cells upon treatment with single therapy. Within two days, a remarkable difference in the peritoneal effusion was noticed upon treatment with the combination. This prompted us to sacrifice the animals after a week of treatment to study the mechanism in detail. ATO/Lena significantly decreased ascites and peritoneal volumes (Figure 1b and Figure S1). Indeed, in mice injected with BC-3 cells, the mean volume of peritoneal ascites decreased from 4 mL in untreated controls, to 2 mL in mice treated with the combination (*p* < 0.01) (Figure 1b). The mean peritoneal volume was also decreased to 40% in ATO/Lena treated mice (Figure S1) (*p* < 0.001). Similarly, in mice injected with BCBL-1 cells, the mean volume of peritoneal ascites decreased from 7 mL in untreated control to 1.4 mL in ATO/Lena-treated mice (*p* < 0.001), and the mean peritoneal volume decreased to 28% in mice treated with the combination (*p* < 0.001) (Figure 1b and Figure S1). Collectively, these results demonstrate that the ATO/Lena combination reduces effusion and enhances survival in PEL mice.

### 2.2. ATO/Lena Inhibits Proliferation and Downregulates KSHV Latent Proteins in Ex Vivo Ascites-Derived PEL Cells 

**Figure 2:**
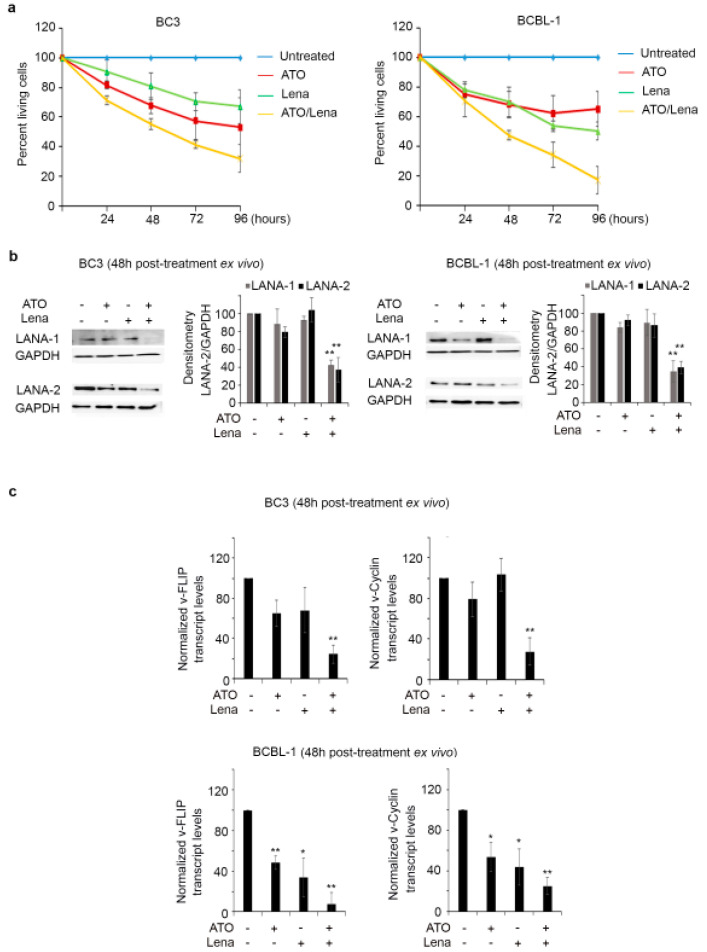
ATO/Lena inhibited proliferation and downregulated Kaposi sarcoma herpes virus (KSHV) latent transcripts and proteins in ex vivo treated ascites-derived BC-3 and BCBL-1 cells. (**a**) Cell proliferation of ascites-derived BC-3 (left) or BCBL-1 cells (right) following ex vivo treatment with ATO and/or Lena for 24, 48, 72, and 96 h. Results are presented as percent of control, plotted as mean ± SD, and represent an average of three independent experiments. (**b**) Immunoblot analysis of KSHV latent proteins LANA-1 and LANA-2 in ascites-derived BC-3 (left) or BCBL-1(right) cells treated ex vivo for 48 h with ATO, Lena, or their combination. Densitometry histograms represent an average of 3 independent experiments. Uncropped blots of Figure 2b are shown in Figure S5 (**c**) Real-time quantitative PCR analysis of transcript levels of KSHV latent genes v-FLIP and v-Cyclin in ascites-derived BC-3 (left) or BCBL-1(right), 48 h post treatment with ATO, Lena, or the ATO/Lena combination. Results represent the average of 3 independent experiments. (*) indicates *p* < 0.05; (**) indicates *p* < 0.01.

BC-3 and BCBL-1 cells derived from malignant peritoneal ascites in PEL mice were treated ex vivo with ATO and/or Lena. A moderate but significant effect on cell proliferation was obtained upon treatment with ATO or Lena single agents, starting 48 h post treatment of both ascites-derived PEL cells (*p* < 0.05). Interestingly, treatment with ATO/Lena resulted in a more pronounced anti-proliferative effect in both BC-3 (*p* < 0.01) and BCBL-1 (*p* < 0.001) at 48 and 72 h post treatment (Figure 2a). Moreover, BCBL-1 ascites-derived cells were more sensitive to the ATO/Lena combination than BC-3 cells (Figure 2a).

To dissect the anti-proliferative effect of ATO/Lena on PEL cells, we first tested the expression of KSHV latent proteins, known to play a key role in PEL oncogenesis [2,17,18,19,20,21]. Interestingly, the ATO/Lena combination resulted in the decreased protein expression of both LANA-1 and LANA-2 proteins, at 48 h post treatment, in both BC-3 and BCBL-1 ascites-derived cells (Figure 2b and Figure S2). Similarly, while no significant effect of a single agent was observed, ATO/Lena significantly decreased KSHV latent v-FLIP and v-Cyclin transcript levels in BC-3 ascites-derived cells (*p* < 0.01) (Figure 2c). In BCBL-1 ascites-derived cells, ATO or Lena single agent treatment yielded a significant, albeit moderate, decrease in v-FLIP and v-Cyclin transcript levels ((*p* < 0.05 and 0.01, respectively). Yet, this decrease was more pronounced upon treatment with the ATO/Lena combination (*p* < 0.01) (Figure 2c). The decrease in cellular proliferation and the reduction of expression of viral latent proteins may explain the enhanced survival and the decrease in peritoneal effusion.

### 2.3. ATO/Lena Decreases Tumor Burden through Inhibiting Autocrine Cytokines in Ex Vivo Ascites-Derived PEL Cells

**Figure 3:**
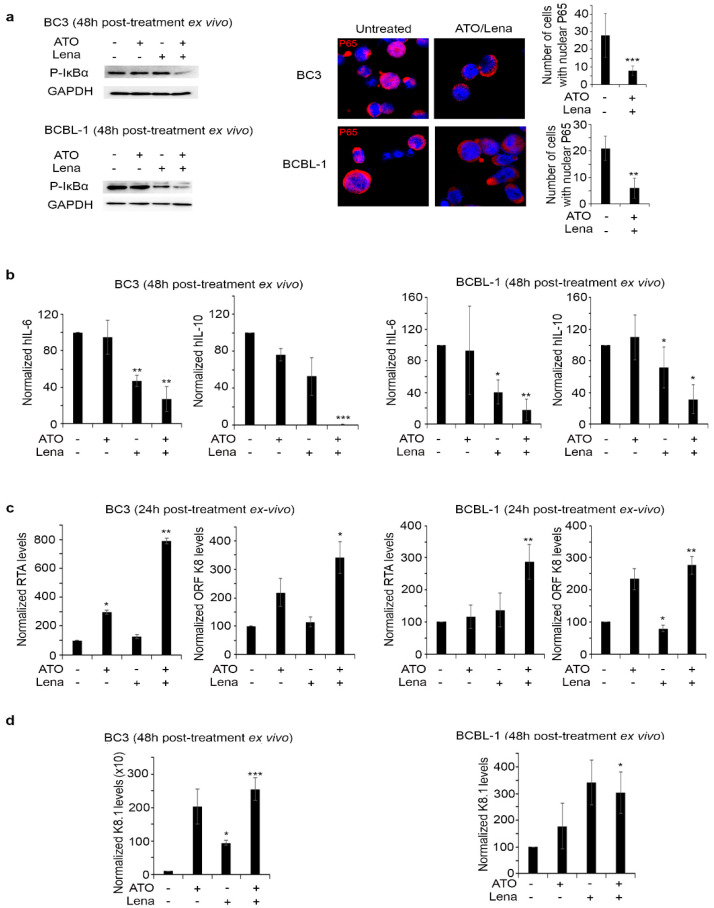
ATO/Lena inhibited NF-κB activation and increased KSHV reactivation in ex vivo treated ascites-derived BC-3 and BCBL-1 cells. (**a**) Western blot analysis of p-IκBα in ATO/Lena ex vivo treated ascites-derived BC-3 and BCBL-1 cells 48 h post treatment. Confocal microscopy analysis of p65 nuclear translocation in ascites-derived BC-3 and BCBL-1 after ex vivo treatment with ATO/Lena for 48 h. p65 was stained with anti-p65 antibody (red) and nuclei were stained by Hoechst stain (blue). Images represent z-sections. Histograms represented number of cells with nuclear p65 translocation. Uncropped blots of Figure 3a are shown in Figure S5. (**b**) Real-time quantitative PCR showing transcript levels of human IL-6 and IL-10 cytokines in ascites-derived BC-3 (left) and BCBL-1 (right) cells 48 h following ex vivo treatment with ATO and/or Lena. (**c**,**d**) Real-time quantitative PCR analysis of transcript levels of KSHV early-lytic genes (RTA, ORFK8) (**c**) or late lytic gene (K8.1) (**d**) in ascites-derived BC-3 (left) and BCBL-1 (right) cells after ex vivo treatment with ATO and/or Lena for 24 (**c**) or 48 h (**d**) as indicated. Results represent the average of 3 independent experiments. (*) indicates *p* < 0.05; (**) indicates *p* < 0.01; and (***) indicates *p* < 0.001.

Prior studies demonstrated an important role of v-FLIP in the activation of NF-κB, a constitutively activated pathway with pivotal roles in PEL progression and survival [29,32,35]. In accordance with the decreased v-FLIP expression, we demonstrated that ATO/Lena decreased phosphorylation of IκBα protein in both BC-3 and BCBL-1 ascites-derived cells at 48 h post treatment (Figure 3a). This was consistent with the decreased nuclear translocation of p65, a mandatory subunit for the activation of NF-κB pathway, in both BC-3 and BCBL-1 cells, while ATO or Lena alone did not display any effect on p65 translocation (Figure 3a).

NF-κB transactivates the expression of several cytokines, including cellular IL-6 and IL-10. In both BC-3 and BCBL-1 ascites-derived cells, ATO as a single agent had no significant effect on cellular IL-6, while Lena alone significantly decreased cellular IL-6 transcripts in both BC-3 (*p* < 0.01) and BCBL-1 cells (*p* < 0.05) to more than 50% as compared to untreated controls (Figure 3b). Single agent treatment with ATO or Lena had no effect on cellular IL-10 transcript levels. Importantly, the ATO/Lena combination significantly decreased IL-6 transcripts to 27% (*p* < 0.01) and totally abrogated IL-10 expression (*p* < 0.001) in BC-3-ascites-derived cells. In BCBL-1 ascites-derived cells, a similar decrease to 18% in cellular IL-6 (*p* < 0.01) and to 31% in cellular IL-10 (*p* < 0.05) was obtained (Figure 3b). Hence, it is conceivable to explain the reduction in cellular proliferation due to the treatment as converging pathways through decreasing NF-κB, leading to the subsequent decrease in the transcription of autocrine cytokines necessary for PEL cell survival.

### 2.4. ATO/Lena Induces KSHV Lytic Gene Expression in Ex Vivo Ascites-Derived PEL Cells

In PEL, NF-κB constitutive activation inhibits KSHV reactivation and lytic gene expression [55,56]. In contrast, KSHV reactivation and increased lytic gene expression is often paralleled with decreased latent transcription and apoptosis [21]. Interestingly, the ATO/Lena combination resulted in significant induction of early RTA and ORFK8 lytic transcript levels at 24 h and late lytic gene transcripts (ORFK8.1) at 48 h post treatment of ex vivo ascites-derived PEL cells (Figure 3c,d). Indeed, in BC-3 ascites-derived cells, ATO alone resulted in a moderate significant increase of RTA transcripts 24 h post treatment (*p* < 0.05), while the Lena single agent had no effect (Figure 3c). The combination of ATO/Lena resulted in a sharp and significant increase in RTA expression 24 h post treatment (*p* = 0.01263). Similar results were observed in ORF K8 transcripts (*p* = 0.0177) upon treatment with the combination for 24 h, while no significant effects of single agents were observed (Figure 3c). The effect of the ATO/Lena combination was similar in BCBL-1 derived ascites, whereby 24 h of treatment resulted in a significant and sharp increase of both early lytic gene transcripts RTA (*p* < 0.05) and ORF K8 (*p* < 0.01) (Figure 3c). We then interrogated the late lytic gene K8.1 expression at 48 h post treatment of ascites-derived PEL cells and demonstrated a significant increase of transcript levels upon treatment with the ATO/Lena combination, in both ascites-derived BC-3 (*p* < 0.01), and BCBL-1 cells (*p* < 0.05) (Figure 3d).

### 2.5. ATO/Lena Induces Apoptosis in Ex Vivo Ascites-Derived PEL Cells

**Figure 4:**
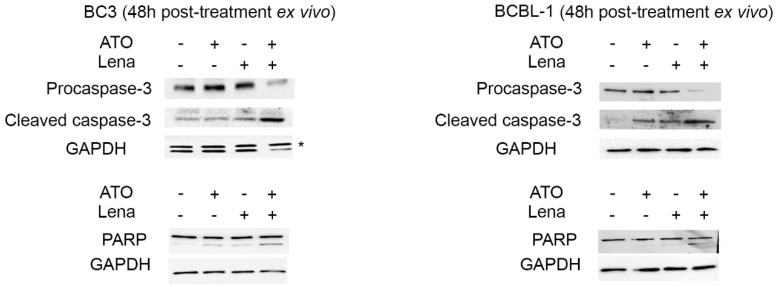
The ATO/Lena combination induced apoptosis in ex vivo treated ascites-derived BC-3 and BCBL-1 cells. Western blot analysis of PARP, procaspase-3, and cleaved caspase-3 levels in ascites-derived BC-3 (left) and BCBL-1 (right) cells 48 h post treatment as indicated. Uncropped blots are shown in Figure S5.

Inhibition of NF-κB induces apoptosis in PEL cells in vitro [57]. In both ascites-derived BC-3 and BCBL-1 cells, the combination of ATO/Lena induced a prominent cleavage of procaspase-3, a crucial mediator of apoptosis, and an increased expression of cleaved caspase-3 protein (Figure 4). Consistent with these results, PARP-1, a downstream target of caspase-3, was also cleaved in both ascites-derived BC-3 and BCBL-1 cells 48 h post treatment with the combination of ATO/Lena (Figure 4), indicating the induction of apoptosis. A similar experiment was also performed with DAPI staining (Figure S3). This allows the evaluation of the percentage of chromatin condensation, which is a hallmark of apoptosis.

### 2.6. ATO/Lena Inhibits VEGF-Dependent Endothelial Cells Tube Formation

**Figure 5:**
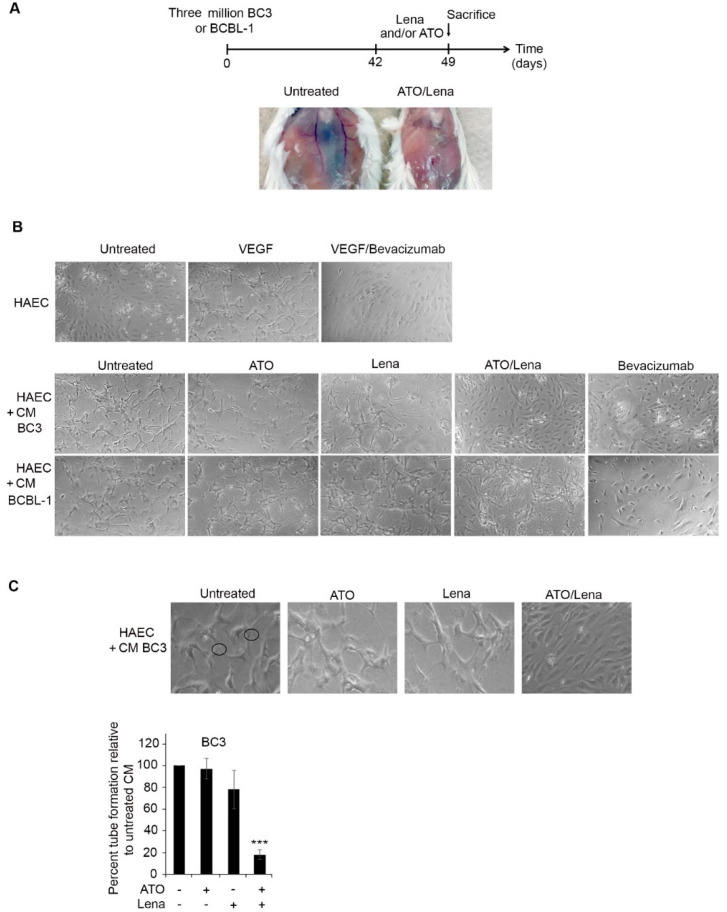
ATO/Lena decreased VEGF-induced tubal formation potential of ascites-derived BC-3 but not BCBL-1 cells. (**A**) Mice peritoneum vascularization before and after one-week treatment with ATO/Lena in BC-3 PEL mice. (**B**) Light microscopy images of capillary-like tube formations in HAEC cells following incubation with supernatants from ex vivo treated ascites-derived BC-3 or BCBL-1 cells. (**C**) Tubal formation analysis method where nodes with 3 or more branches were counted and compared. At least 5 images from each condition were counted. Data was reported as a histogram of the percentage of tube formation relative to untreated ascites-derived BC-3 cells. Data represent an average of 3 independent experiments. (***) indicates *p* < 0.001.

In BC-3 injected mice, we noticed a decreased peritoneal vascularization upon treatment with the combination of ATO/Lena (Figure 5A). The anti-angiogenic effect of Lena was previously documented in numerous blood malignancies [58,59]. PEL cells are also known to secrete VEGF into ascitic fluids in vivo, promoting VEGF-dependent angiogenesis and PEL progression [60,61]. We investigated the effect of ATO/Lena on VEGF-dependent angiogenesis. HAECs were seeded onto growth factor-reduced Matrigel and incubated with treated or untreated ascites-derived PEL (BC-3 or BCBL-1) cell-free supernatant for 48 h. When cultured alone, HAEC cells failed to form structured tubes. In contrast, the addition of supernatants from untreated ascites-derived BC-3 and BCBL-1 cells resulted in capillary-like tubes with multi-centric junctions (Figure 5B). Supernatant from ATO or Lena treated BC-3 or BCBL-1 cells had no or minimal effects on tubal formation capacity. In contrast, supernatant from ascites of ATO/Lena treated BC-3 PEL mice resulted in a significant reduction in the capillary-like tube formation capacity compared to untreated cells, while no effect of this combination was observed in BCBL-1 PEL mice, presumably due to either the relatively low amount of Bevacizumab or the overwhelming amount of VEGF secreted by BCBL-1 [61]. This suggests that reduction in VEGF-induced angiogenesis may be one of the mechanisms contributing to ATO/Lena eradication of ascites-derived BC-3 but not BCBL-1 cells.

### 2.7. ATO/Lena Decreases Organ Infiltration and Downregulates Latent KSHV Proteins and Cytokine Expression In Vivo

**Figure 6:**
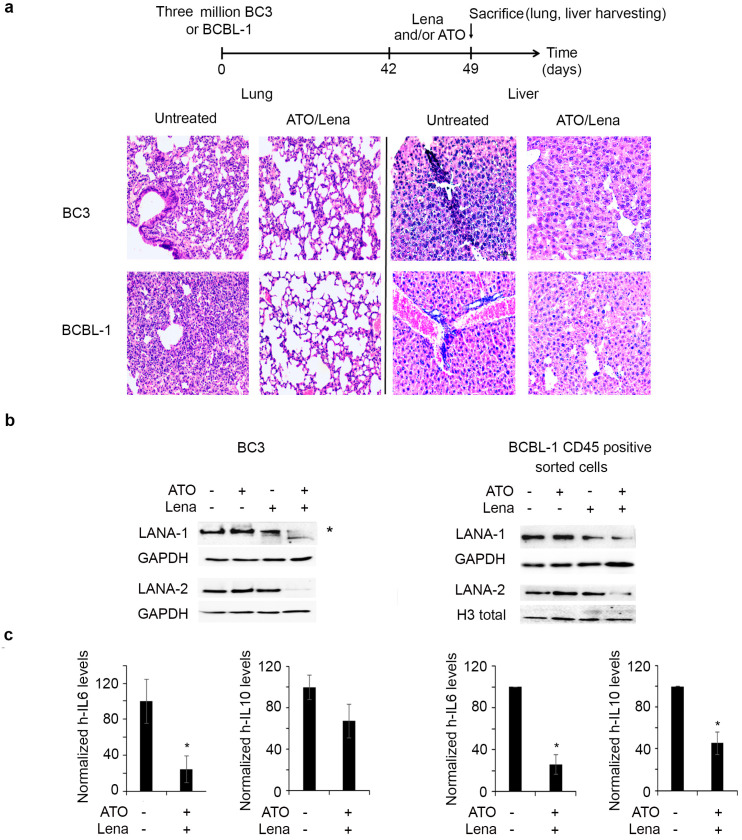
ATO/Lena decreased organ infiltration and downregulated latent KSHV proteins in vivo. (**a**) Experimental design: mice injected with BC-3 or BCBL-1 were allowed to develop ascites for 6 weeks, were treated with ATO and/or Lena for one week, then were sacrificed. Histopathology sections of lung and spleens from ATO/Lena treated or untreated BC-3 and BCBL-1 PEL mice. (**b**). Immunoblot analysis of LANA-1 and LANA-2 proteins in whole ascites (left) or CD45+ sorted ascites (right) derived from treated or untreated BC-3 and BCBL-1 mice respectively. Uncropped blots of Figure 6b are shown in Figure S5. (**c**) Real-time quantitative PCR analysis of cellular IL-6 and IL-10 transcript levels in lungs of ATO/Lena treated or untreated BC-3 and BCBL-1 mice as indicated. Transcript levels were normalized to GAPDH internal levels. (*) indicates *p* < 0.05

To investigate the in vivo effect and mechanism of action of the ATO/Lena combination, PEL mice were left to form lymphomatous effusion for 6 weeks before treatment for one week with the ATO/Lena combination. We first examined the effect of this therapeutic regimen on organ infiltration. Compared to control mice, ATO/Lena treated mice exhibited decreased infiltration of PEL cells in lungs and livers of both BC-3 and BCBL-1 models (Figure 6a). Consistent with ex vivo data, ATO/Lena treatment decreased LANA-1 and LANA-2 protein expression in effusions of treated BC-3 and BCBL-1 mice. We then questioned whether this decrease was merely a result of decreased percentage of PEL cells in collected ascites. CD45 staining of ascites from ATO/Lena BC-3 treated mice showed no significant variation in the number of PEL cells as compared to untreated mice (Figure S4). Thus, the obtained decrease in LANA-1 and LANA-2 proteins upon treatment with ATO/Lena is not due to less PEL cells, but rather an effect of this combination on viral protein expression (Figure 6b, left panel). In BCBL-1 mice, treatment with ATO, Lena, or their combination affected the number of peritoneal PEL cells, with a significant reduction by 50% in the percentage of CD45 positive cells in ATO/Lena treated mice (Figure S4). We thus sorted CD45 positive BCBL-1 cells before assessing the effect of the combination on viral protein expression in BCBL-1 ascites-derived cells, and we demonstrated that the ATO/Lena combination also decreased LANA-1 and LANA-2 protein expression in these cells (Figure 6b, right panel). We also observed a decreased transcript expression of both cellular IL-6 and IL-10 in the lungs of ATO/Lena treated PEL mice. The decrease of IL-6 was significant in the BC-3 model and reached 25% (*p* < 0.05), while the decrease of both IL-6 and IL-10 was significant in the BCBL-1 model and reached 25% (*p* < 0.05) and 45% (*p* < 0.05), respectively. Overall, these data validate the above ex vivo dissected mechanism of action of the ATO/Lena combination in vivo.

## 3. Discussion

PEL has a dismal prognosis due to significant challenges that include chemo-resistance, lack of large-scale randomized clinical trials, and high relapse rates. Currently, chemotherapy remains the cornerstone treatment for managing PEL, yet, the overall survival rate is limited to a year in only 40% of CHOP-treated patients [11,40]. The combination of ATO and IFN induced cell death and enhanced survival in PEL mice [53]. Despite its significant effect, this combination did not result in a cure. Administration of Lena alone to a PEL patient led to complete and long lasting remission [48]. In addition, a phase I/II clinical study is being performed investigating Lena with chemotherapy and rituximab (NCT02911142). Collectively, this guided our current investigation. The combination of ATO with different agents proved efficient in several blood malignancies. The combination of ATO with retinoic acid became the standard clinical regimen to cure acute promyelocytic leukemia [49]. Similarly, this combination resulted in selective cell death of acute myeloid leukemia with nucleophomin-1 mutation [62,63] and significantly reduced leukemic blasts in patients [62]. In adult T cell leukemia (ATL), ATO synergized with IFNα to selectively induce apoptosis of ATL cells [64], cured the disease in a murine ATL model [65], and yielded a high remission rate in newly diagnosed chronic ATL patients [66]. In PEL, ATO/IFNα inhibited growth, induced apoptosis, and downregulated the latent viral transcripts in PEL cells derived from malignant ascites [53]. In a murine PEL model, this combination decreased the volume of peritoneal ascites, a feature imposing difficulty in treatment of several malignant lymphomatous effusions, and synergistically increased survival of PEL mice [53]. The ATO/Lena combination has been previously investigated in multiple myeloma. ATO and Lena exhibited independent non-interfering effects [67] where ATO sensitized myeloma cells to Lena via the upregulation of cereblon expression, a major target for Lena, resulting in apoptosis [68]. In a different study, the use of a supra-pharmacological dose of Lena (50 mg/kg/day) in PEL mice induced apoptosis of PEL cell lines and decreased ascites formation in vivo [46]. In contrast, our data showed that treatment of ex vivo treated BC-3 or BCBL-1 ascites-derived PEL cells with Lena alone moderately inhibited proliferation and did not decrease peritoneal volume in vivo (Figure 1b and Figure 2a), presumably due to the 10-fold lower concentration used in our experiments. Yet, Lena alone increased survival of BC-3 injected mice and led to a cure in 25% of BCBL-1 injected mice (Figure 1a). While ATO yielded better survival outcome in BC-3 injected mice than did Lena alone, Lena as a single agent showed a better effect on survival on BCBL-1 injected mice (Figure 1a). Yet, neither Lena nor ATO single agents exhibited any effect on peritoneal ascites volume (Figure 1b). Interestingly, ATO/Lena impeded ascites formation, decreased organ infiltration (Figure 1b and Figure 6a), enhanced survival, and induced a cure in 25% and 75% of BC-3 and BCBL-1 PEL mice, respectively (Figure 1a).

KSHV latency is crucial for development of its associated malignancies [17]. The latent state of the virus still presents a substantial hurdle through promoting PEL cell proliferation and survival [17]. KSHV persists in a latent state [21] in PEL cell lines and ascites. Interestingly, ATO/Lena decreased expression of LANA-1 and LANA-2 latent proteins in malignant ascites-derived cells and in vivo (Figure 2b, Figure 6b and Figure S2). Two other latent KSHV proteins, v-Cyclin and v-FLIP, are transcribed from the same promoter [69], yet an internal ribosome entry site preceding the v-FLIP start codon and overlapping the v-Cyclin coding region allows for v-FLIP translation [70]. The decreased expression of all tested latent viral genes (LANA-1, LANA-2, v-Cyclin, and v-FLIP) was consistent in both ascites-derived PEL cells (Figure 2c). v-FLIP is essential for survival of PEL cells, and its silencing results in apoptosis [29]. Moreover, v-FLIP expression in rodent cells resulted in increased proliferation and transformation, highlighting the v-FLIP oncogenic capacity [32]. The mechanism through which ATO/Lena decreased latent protein expression requires further investigation. One potential mechanism can be through the accumulation of reactive oxygen species (ROS) in PEL cells. This may result in decreased latency, viral reactivation, and apoptosis induction [71,72]. Indeed, both ATO and Lena treatments used as single agents are known to induce oxidative stress and ROS generation [52,73,74]. In multiple myeloma, Lena inhibited hydrogen peroxide decomposition, resulting in increased oxidative stress and cytotoxicity [73]. Furthermore, ROS accumulation in target cells is documented as one of the established mechanisms contributing to ATO cytotoxicity [74].

NF-κB activation is involved in KSHV transformation, resistance to apoptosis, and is paramount for survival of PEL cells [33,35,75]. Previous studies presented NF-κB as a potential target for therapy [35,57]. NF-κB inhibition prevented or delayed PEL growth and prolonged disease-free survival in mice [35]. Downregulation of v-FLIP activity resulted in decreased NF-κB activation and subsequent apoptosis of PEL in vitro [29]. Consistent with these reports, we demonstrate that the ATO/Lena decreased v-FLIP expression was accompanied by the inhibition of the NF-κB pathway (Figure 3a). PEL cells are known to produce IL-6 and IL-10 [36,37]. Indeed, v-FLIP is known to activate the IL-6 promoter through NF-κB activation [76]. On the other hand, IL-6 and IL-10 are both downstream NF-κB signalers. These two cytokines are known as autocrine growth factors, which promote PEL growth and proliferation [36,37,38]. Clinically, IL-6 and IL-10 were shown to be prognostic factors that contribute to clinical response and pathogenesis of PEL [77]. In accordance with these studies, NF-κB inhibition following ATO/Lena treatment was accompanied by decreased IL-6 and IL-10 transcripts ex vivo and in vivo (Figure 3b and 6c). The decrease in latent viral proteins expression along with the decrease in essential autocrine factors might explain the pronounced inhibition of cell proliferation inflicted by the ATO/Lena combination.

Suppression of viral lytic gene expression is a fundamental mechanism by which latency is maintained in PEL [17,21]. Latent proteins inhibit the KSHV lytic state and promote oncogenesis. In that sense, LANA-1 suppresses lytic gene expression by binding to gene promoters to inhibit transcription or via epigenetic silencing of the KSHV genome [19]. v-FLIP inhibits RTA promoter activity, a major lytic transactivator, and represses viral lytic replication [76]. v-FLIP mediated inhibition of lytic reactivation occurs through activation of the NF-κB pathway and inhibition of AP-1 [76]. In fact, deletion of v-FLIP increased expression of lytic genes and enhanced lytic replication [76]. Recently, it was shown that NF-κB inhibition resulted in KSHV reactivation and cell death [72]. Moreover, KSHV reactivation was proposed as a potential therapeutic approach for eradicating PEL [78]. In line with these data, and concomitant with the decreased latent viral expression and inhibition of NF-κB and its subsequent decrease of IL-6 and IL-10, ATO/Lena induced an increase in the transcription of both the early viral lytic genes (RTA and ORF K8) and the late viral lytic gene (K8.1) (Figure 3c,d) and the induction of apoptosis (Figure 3b and Figure 4).

High levels of VEGF are secreted by PEL cells [60] and correlate with PEL progression [60,61]. Furthermore, anti-VEGF antibodies impede ascites formation in PEL mice [38,60]. The peritoneum of PEL mice exhibited extensive vessel formation (Figure 5a). In animals treated with ATO/Lena, there was a remarkable decrease in abdominal vascularization in BC-3 treated PEL mice (Figure 5a). This was correlated with decreased tube formation capacity in the supernatant of ascites-derived BC-3 cells treated with ATO/Lena (Figure 5b,c). In ascites-derived BCBL-1 cells, basal secreted VEGF levels were much higher compared to ascites derived BC-3 cells [61]. The inability of the antibody to inhibit tube formation can be explained by the very high levels of VEGF in those cells [61]. 

Our study provides coherent evidence on the efficacy of ATO/Lena against PEL in vivo and ex vivo, which strongly supports the clinical testing of this combination for a better management of PEL.

## 4. Materials and Methods

### 4.1. Cells, Mice, and Treatment

The KSHV^+^/EBV^-^ BC-3 and BCBL-1 malignant B cell lines derived from PEL patients [79,80] were obtained from Dr A. Gessain (Pasteur Institute, Paris, France). Cells were cultured in RPMI-1640 medium containing 10% heat inactivated fetal bovine serum (FBS) and antibiotics. Six to eight week old immuno-compromised male or female NOD/SCID mice (Charles River, Ecully, France) were inoculated with 2 million BC-3 or BCBL-1 cells [81]. Starting at day 4 post-PEL inoculation, mice were injected intraperitoneally daily with ATO (Sigma Aldrich, Sigma Aldrich, MO, USA, cat#A1010) and/or Lena (Celgene Corporation Research Alliance, Paris, France) at the dose of 5 μg/g/day [53] until day 42. None of the individual or combination treatment regimens were toxic in normal NOD/SCID mice when given for 21 days (100% survival for >3 months; no observed toxicity)). Mice survival curves were presented using the Kaplan–Meier method. Statistical analysis (*n* = 4 per condition) was performed using GraphPad prism software 7.0 (GraphPad software ©, San Diego, CA, USA); a *p* value of 0.05 was considered significant.

For therapeutic efficacy, mice were first allowed to develop PEL for 6 weeks and then were treated with ATO and/or Lena at the dose of 5 μg/g/day for one week. To assess ascites development, PEL NOD/SCID (PEL mice) (*n* = 8) were visually monitored and peritoneal diameter (d) was measured using a caliber. Peritoneal volume (indicative of effusion) was calculated according to the formula: v = 4/3π (d/2) [53,82]. Statistical analysis was performed using GraphPad prism software 7.0 and one way Anova. A *p* value of 0.05 was considered significant. Lungs and livers from treated or untreated mice were fixed, embedded in paraffin, sectioned, stained with hematoxylin and eosin (H&E), and examined by light microscopy.

Animal protocols were approved by the Institutional Animal Care and Utilization Committee (IACUC) of the American University of Beirut (AUB) under Permit Number #15-07-P575, and mice were housed in pathogen-free facilities.

Human specific cell surface marker (CD45 phycoerythrin antibody (BD biosciences, San Jose, CA, USA) was performed on cells collected from ascites. Labeled samples were analyzed on a Guava flow cytometer (Merk Millipore, Darmstadt, Germany). Cell sorting was performed using a BD FACSAria SORB, San Jose, CA, USA).

### 4.2. Ex Vivo Cell Culture and Analysis

BC-3 and BCBL-1 were collected from ascites of PEL mice six weeks post-inoculation of cells. Cells were then cultured at the density of 2 × 10^5^ cells per mL in RPMI-1640 medium with 10% FBS and antibiotics. Cells were treated with 1 μM ATO and/or 0.5 μM Lena for 24, 48, 72, or 96 h. Cell growth was assessed using trypan blue exclusion dye assay.

### 4.3. Gene Expression Studies

Total RNA from lungs of treated or untreated PEL mice or from ex vivo treated PEL cells was extracted using Trizol (Qiagen, Hilden, Germany Cat# 79306). cDNA synthesis was performed using a Revert Aid First cDNA synthesis Kit (Thermo Scientific, Waltham, MA, USA). Syber green qRT PCR was performed using the BIORAD CFX96 machine, and the primers for qRT-PCR are listed in Table S1. In qRT-PCR, individual reactions were prepared with 0.25 µM of each primer, 150 ng of cDNA, and SYBR Green PCR Master Mix to a final volume of 10 μL. PCR reactions consisted of a DNA denaturation step at 95 °C for 3 min, followed by 39 cycles of denaturation at 95 °C for 15 s, annealing at 57 °C for 60 s, extension at 72 °C for 30 s. For each experiment, reactions were performed in duplicate and expression of individual genes was normalized to the housekeeping gene glyceraldehyde-3-phosphate dehydrogenase (GAPDH) (Table S1). The transcript expression level was calculated according to the Livak method [83].

### 4.4. Protein Expression Studies

Ex vivo treated PEL cells were solubilized in lysis buffer. One hundred µg of protein lysates were loaded onto a 12% SDS-polyacrylamide gel, subjected to electrophoresis, and transferred onto nitrocellulose membranes. Blots were incubated overnight with specific primary antibodies against LANA-1 (NBP1-30176, Novus), LANA-2 (NB200-167H, Novus), Caspase 3 (sc-7148, Santa Cruz), PARP (sc-7150, Santa Cruz), p-IκBα (Invitrogen, Foster City, CA, USA)., H3 total (ab1791, Abcam, Cambridge, UK), and GAPDH (MAB5476, Abnova, Walnut, CA, USA). Blots were then incubated with appropriate HRP-conjugated secondary antibodies (m-IgGK BP-HRP sc-516102, mouse anti-rabbit IgG-HRP sc-2357, Santa Cruz, CA, USA). Bands were visualized by chemiluminescence (Clarity max, Bio-Rad, Hercules, CA, USA Cat# 170-5061).

### 4.5. Immunofluorescence Assay

Ex vivo untreated or treated PEL cells were fixed with ice-cold methanol (−20 °C) for 20 min and cytospun onto glass slides. Following permeabilization, immunostaining was performed overnight with primary rabbit monoclonal antibody against P65 (Cell Signaling, MA, USA) and rat monoclonal antibody against LANA-1 (Abcam). Primary antibodies were revealed by Alexa Fluor 488- or Fluor 594-labeled secondary antibodies (Abcam). Staining of nuclei was performed with Hoechst (Invitrogen), and slides were mounted using prolong antifade (Invitrogen). For the apoptosis assay, cells were fixed with methanol and stained with Hoechst. Images were acquired by confocal microscopy using a Zeiss LSM710 confocal microscope (Zeiss, Oberkochen, Germany) with a Plan Apochromat 63/1.4 numeric aperture oil-immersion objective using Zen 2009 (Carl Zeiss, Oberkochen, Germany).

### 4.6. Matrigel-Induced Capillary Tube Formation

Twenty-four-well plates were pre-coated with 200 μL of gelled growth factor-reduced Matrigel (Becton Dickinson, San Jose, CA, USA). Human aortic endothelial cells (HAEC) were seeded at the density of 8 × 10^4^ cells for 18 h. Ascites-derived BC-3 or BCBL-1 PEL cells were pelleted and cultured for 48 h in serum-free media. The resulting supernatants from treated or untreated cells were added for 48 h. VEGF was used as a positive control and Bivacizumab to neutralize VEGF as a negative control [84]. Plates were photographed using a Zeiss light microscope and analyzed using Zeiss Zen software. Quantification was performed by counting the nodes, defined as joint point of 3 or more branches [85]. Data was presented as percentage of control/untreated cells.

## 5. Conclusions

Our study depicts ATO/Lena as a promising therapeutic target for PEL. Current PEL regimen relies on chemotherapy, which fails to maintain satisfactory clinical response. Thus, PEL is associated with a dismal prognosis and high relapse rate.

ATO/Lena resulted in enhanced survival and cured PEL mice in vivo. The mechanism of action of ATO/Lena was unveiled: ATO/Lena decreased expression of latent viral proteins, decreased NF-κB activation and cytokine production, and increased lytic gene expression. All of which contributed to induction of apoptosis. Our study warrants the further clinical investigation of the efficacy of ATO/Lena in PEL patients.

## Supplementary Materials

The following are available online at https://www.mdpi.com/2072-6694/12/9/2483/s1, Figure S1: ATO/Lena decreased peritoneal volume in PEL mice. Figure S2: ATO/Lena decreased LANA-1 expression in ascites-derived BC-3 and BCBL-1 PEL cells. Figure S3: ATO/Lena induced apoptosis in ascites-derived BC-3 and BCBL-1 PEL cells, Figure S4: CD45 staining of peritoneal ascites from PEL mice, Figure S5: Uncropped Western blot figures. Table S1: List of primers used for RT-qPCR.
