# Supplementary material for: Lenalidomide in Combination with Arsenic Trioxide: an Effective Therapy for Primary Effusion Lymphoma
Source: Cancers (Basel). 2020 Sep 1;12(9):2483. doi: 10.3390/cancers12092483 (PMC7563318; doi:10.3390/cancers12092483)

Supplementary fig. 5 wb

Supplementary of Fig. 2B

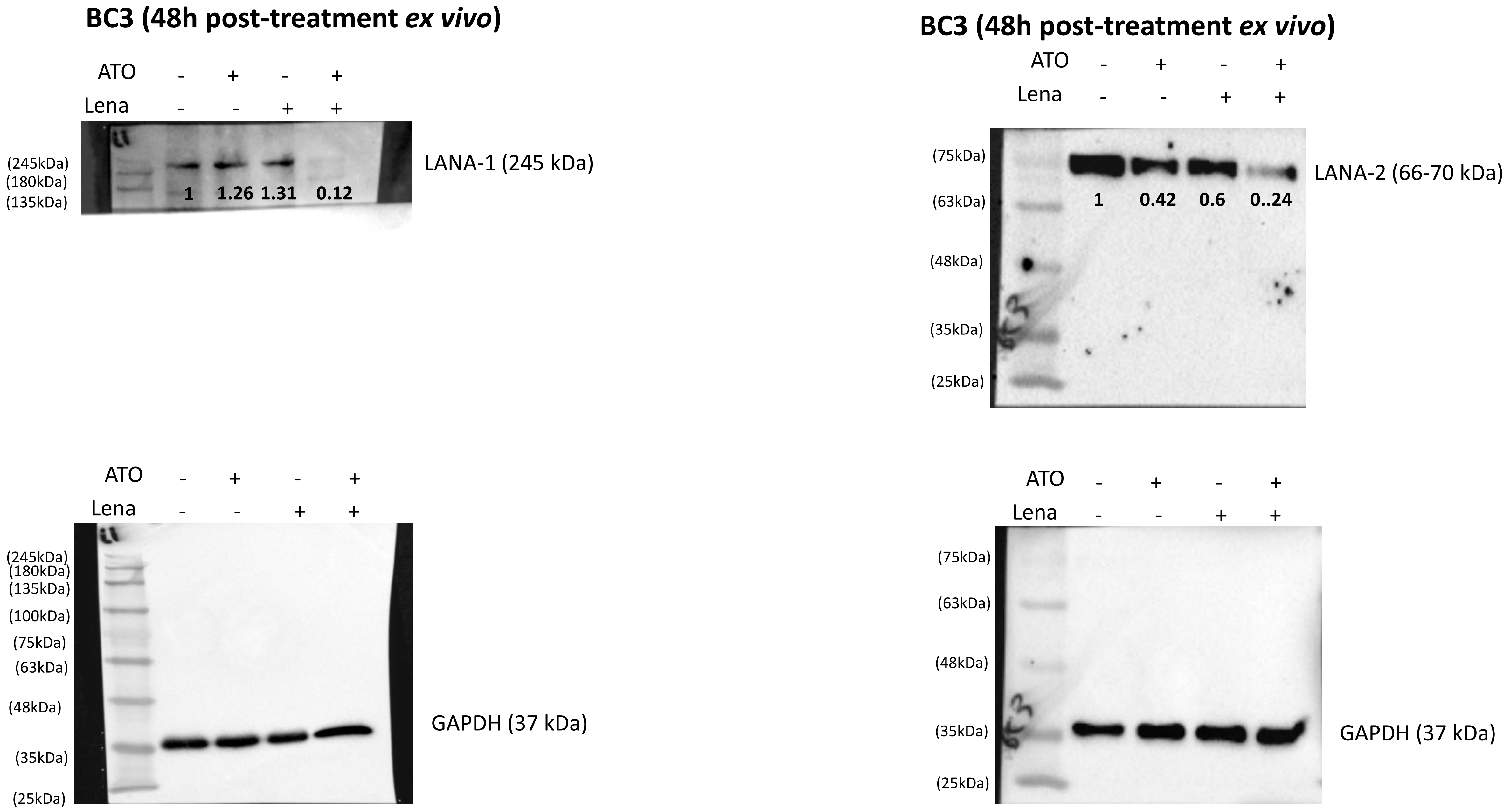

Supplementary of Fig. 2B

BCBL-1 (48h post-treatment *ex vivo*)

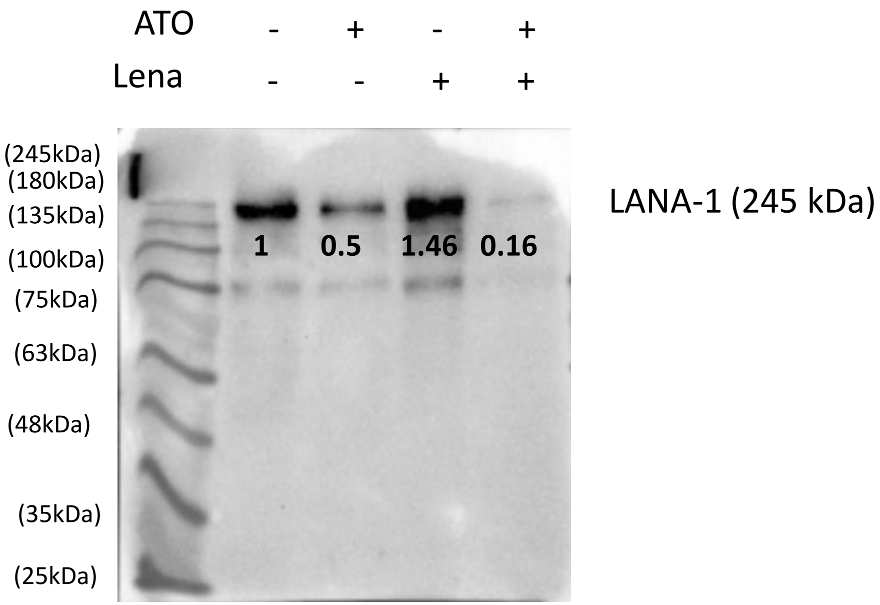

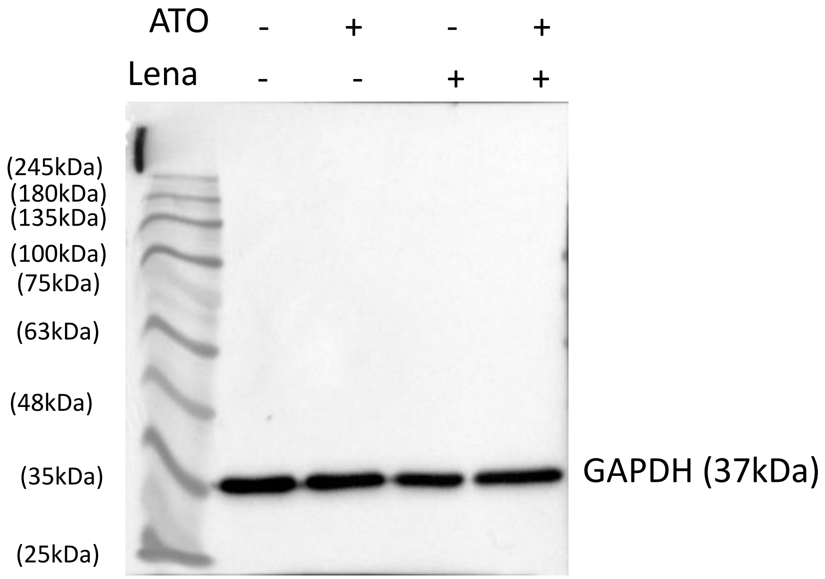

BCBL-1 (48h post-treatment *ex vivo*)

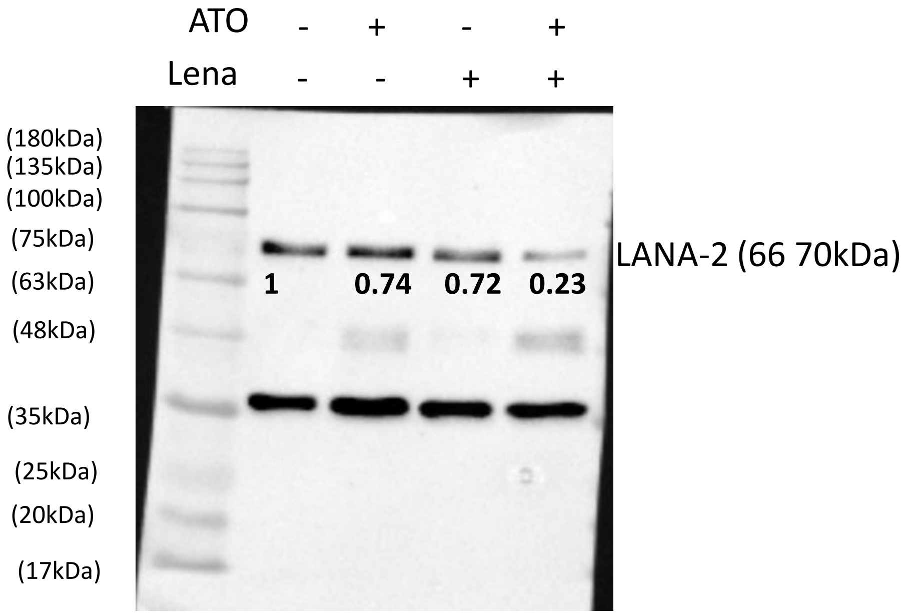

Supplementary of Fig. 3A

BC-3 (48h post-treatment *ex vivo*)

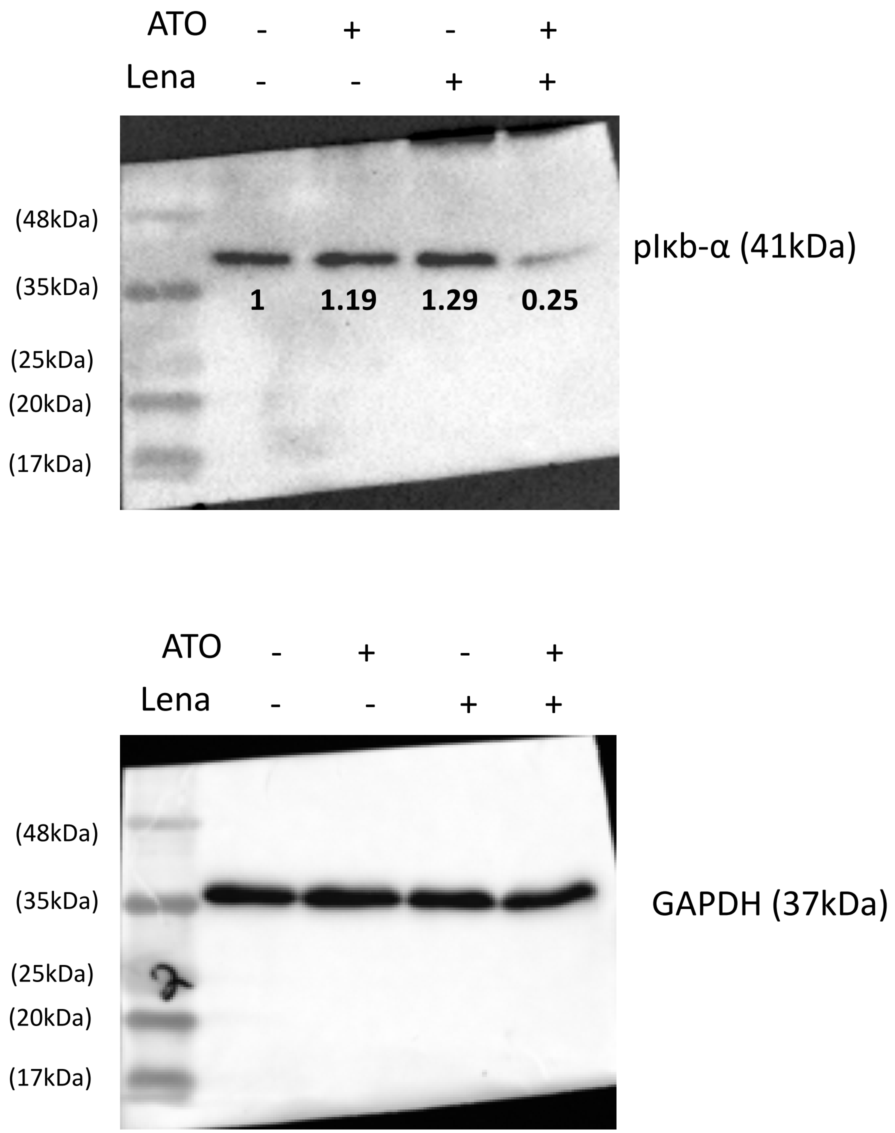

BCBL-1 (48h post-treatment *ex vivo*)

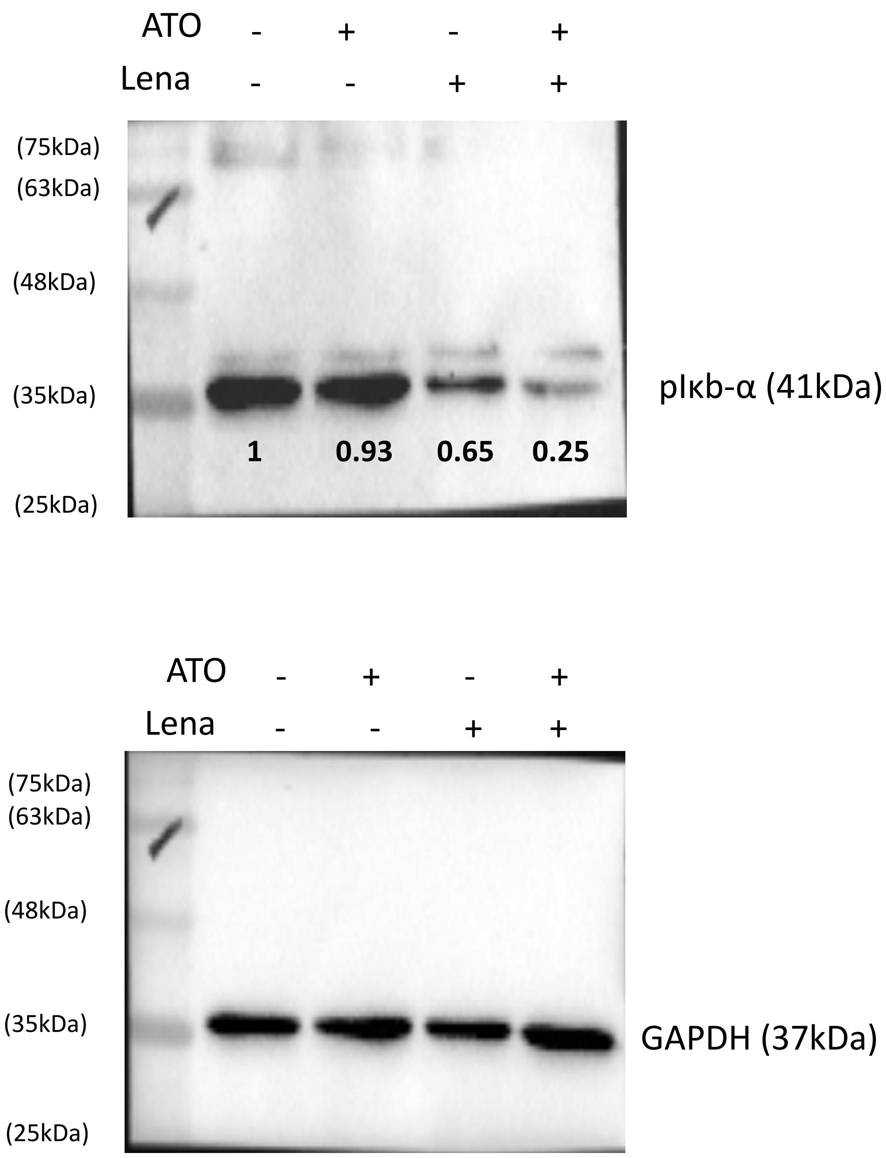

Supplementary of Fig. 4A

BC-3 (48h post-treatment *ex vivo*)

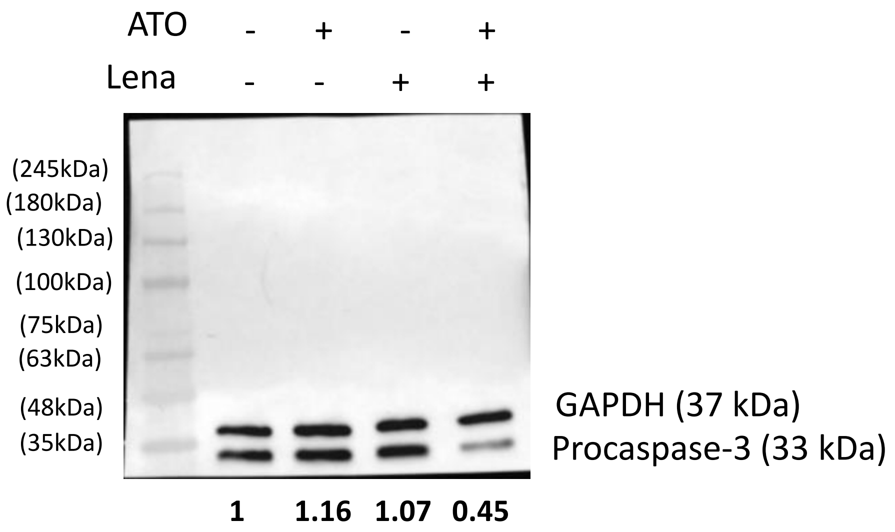

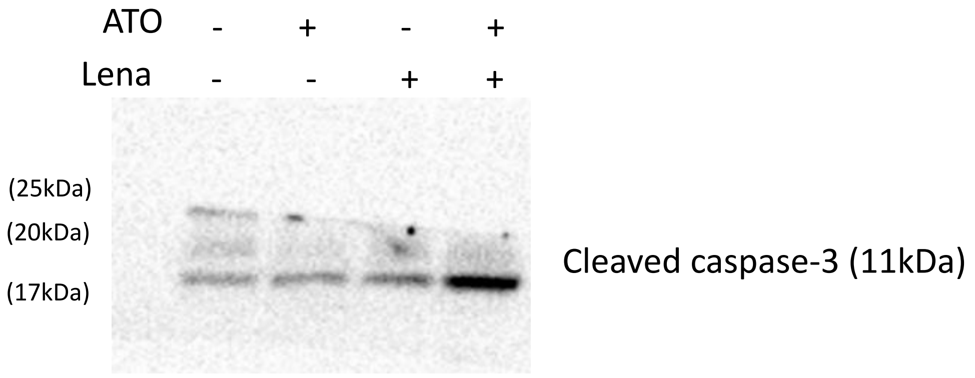

BC-3(48h post-treatment *ex vivo*)

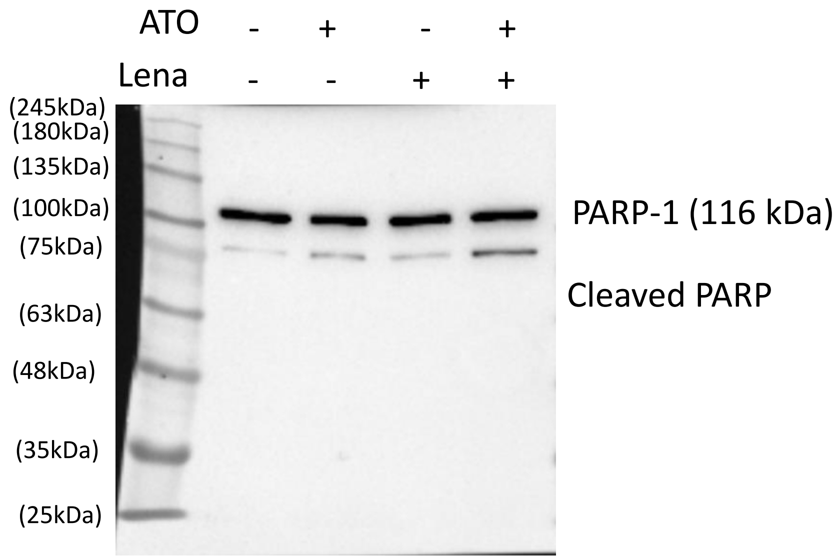

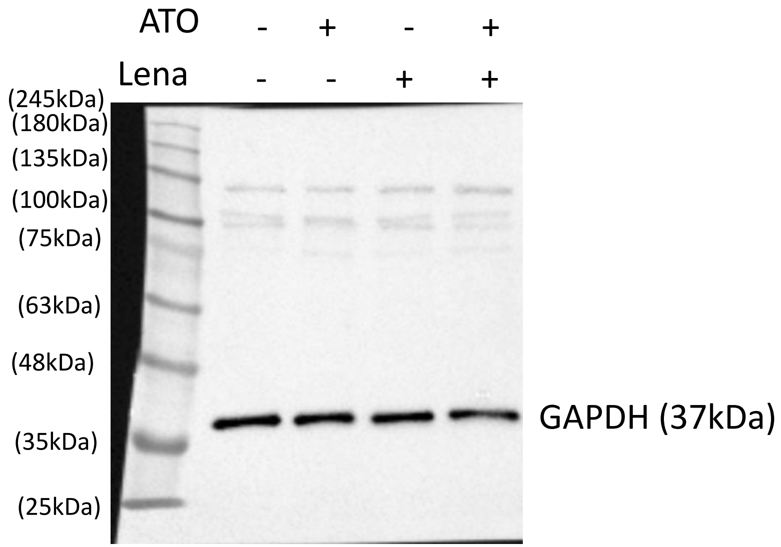

Supplementary of Fig. 4A

BCBL-1 (48h post-treatment *ex vivo*)

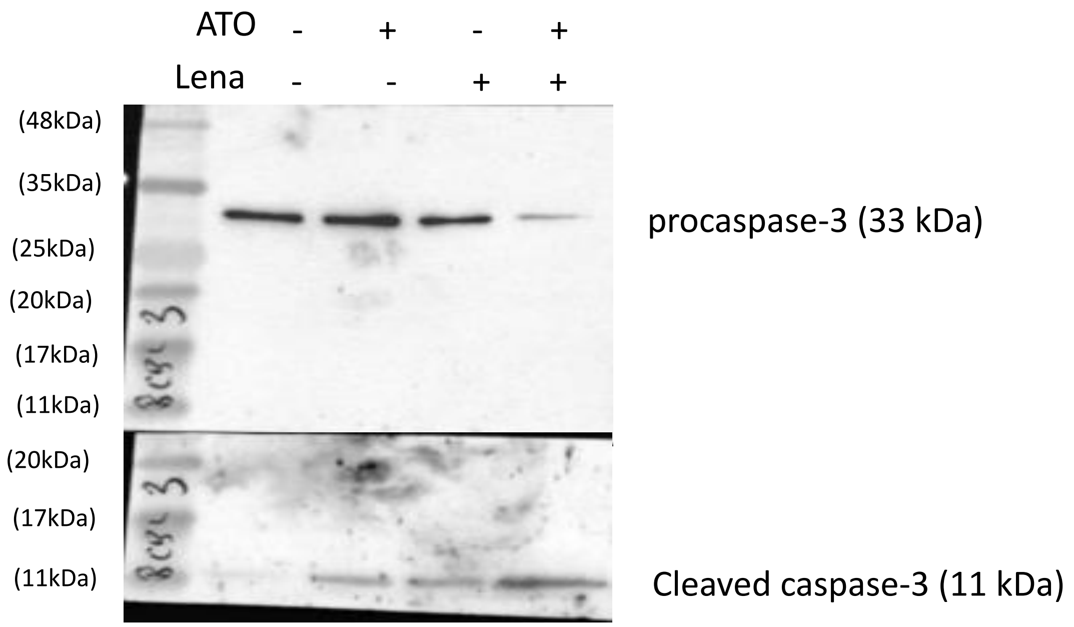

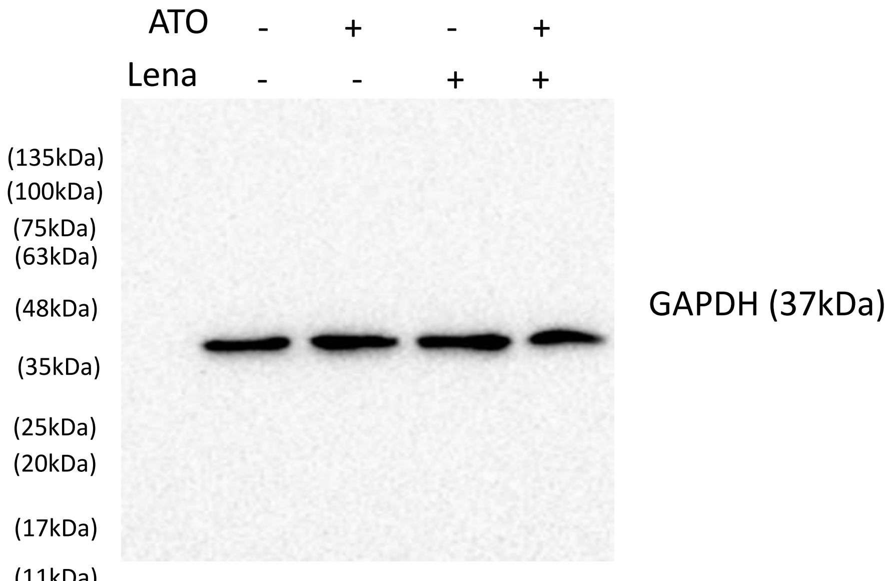

BCBL-1 (48h post-treatment *ex vivo*)

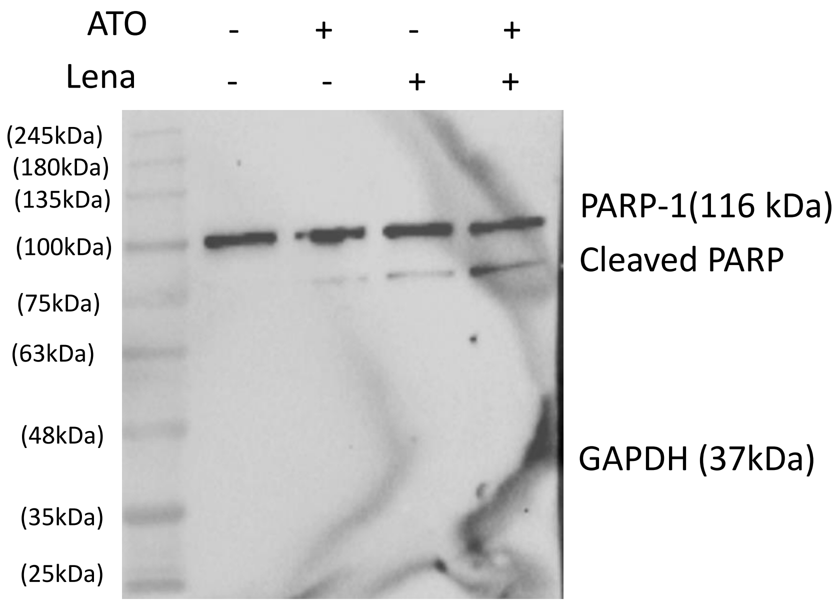

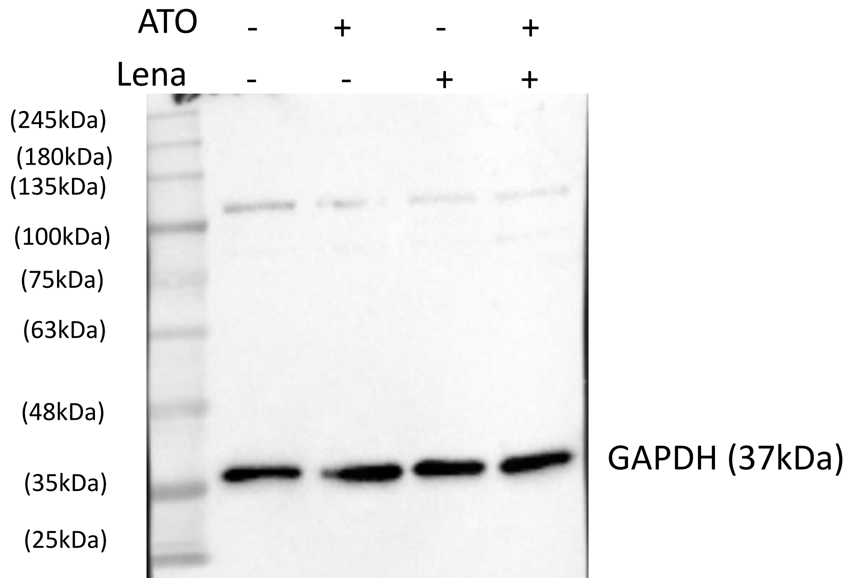

Supplementary of Fig. 6B

BC-3 (*in vivo*)

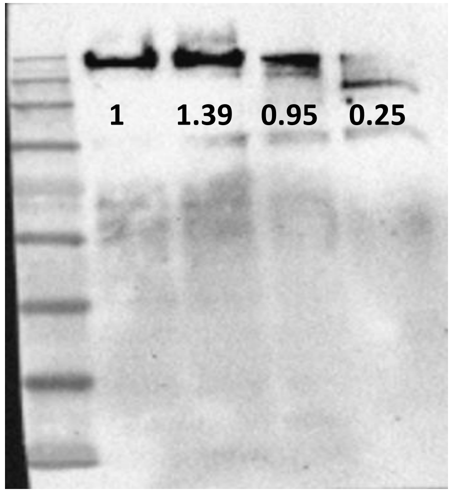

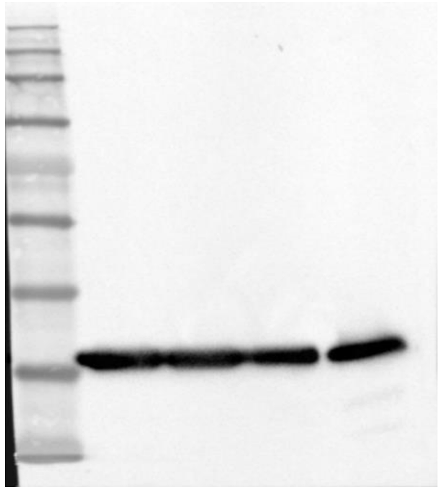

BC-3 (*in vivo*)

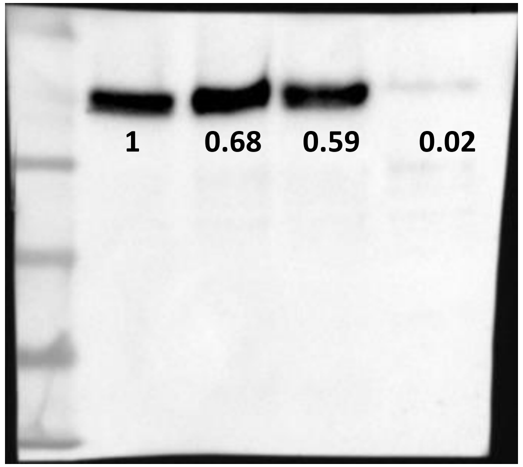

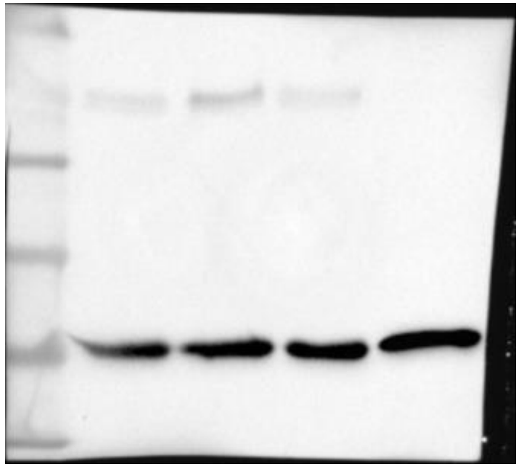

Supplementary of Fig. 6B

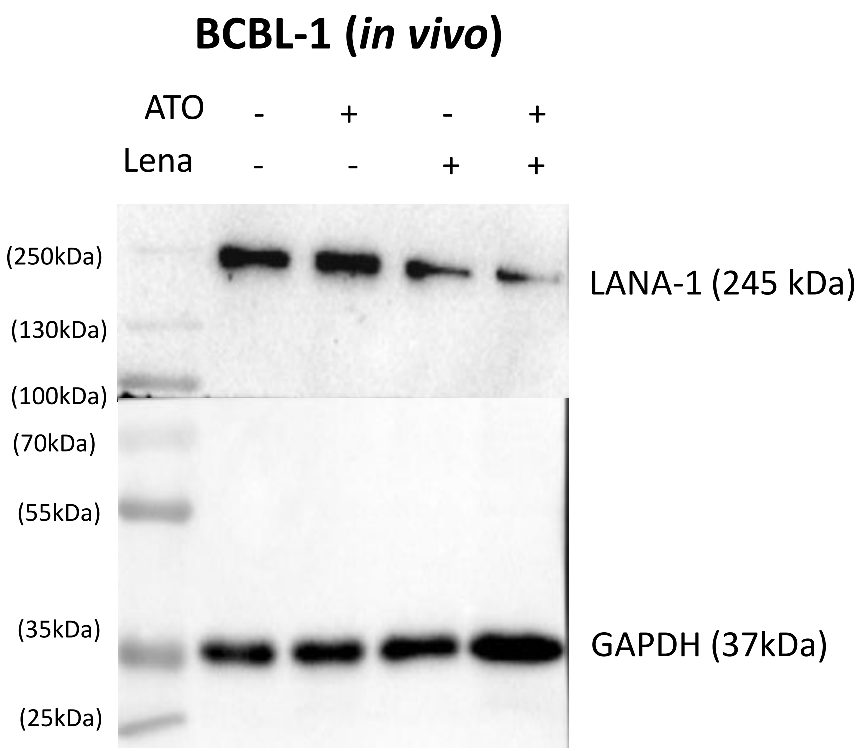

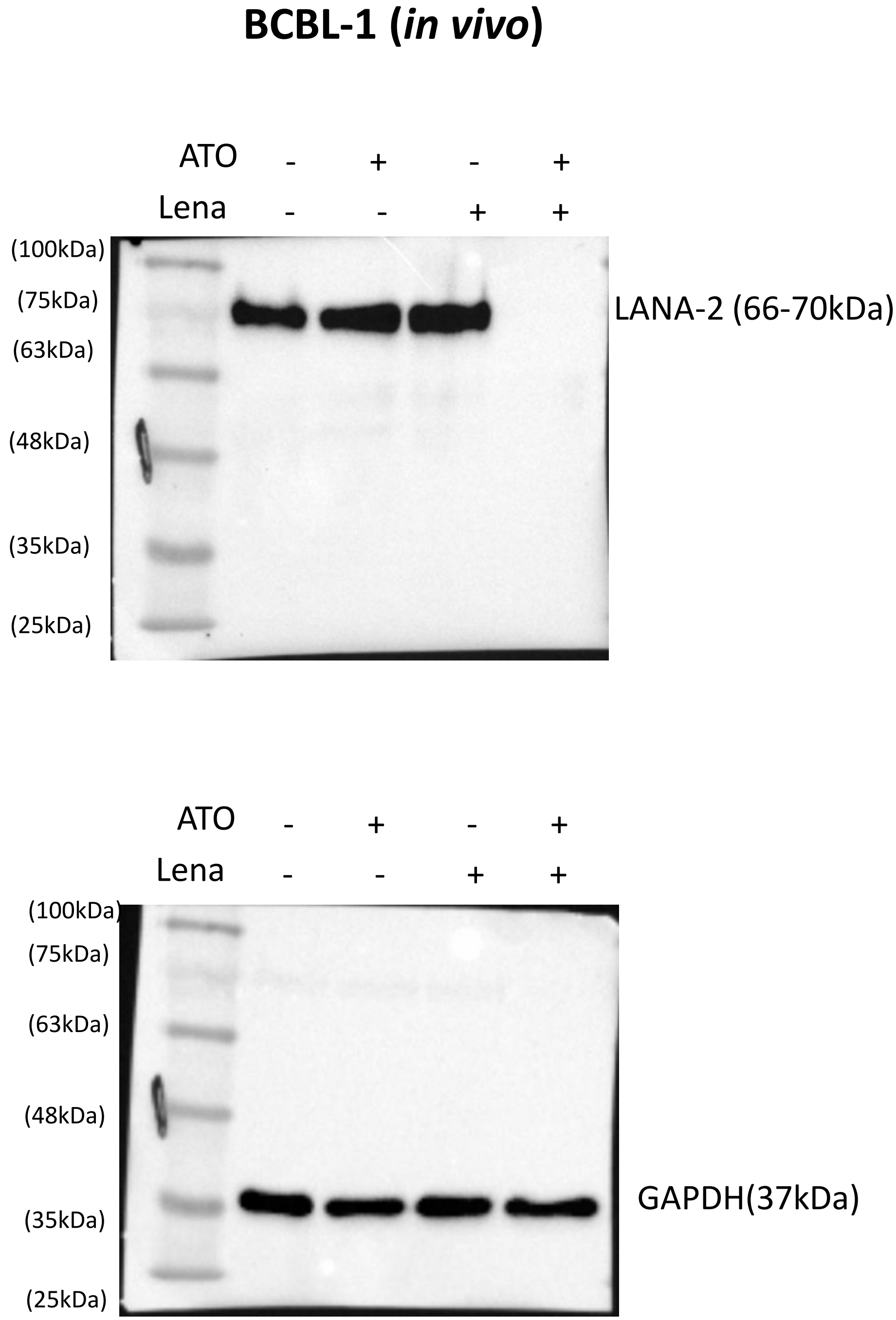

Supplement: Supplementary file 1 [file cancers-12-02483-s001.zip › cancers-871644 supplementary file/cancers-871644-Figure S5-Uncropped Western blots figures.pdf]
