# Supplementary material for: Lenalidomide in Combination with Arsenic Trioxide: an Effective Therapy for Primary Effusion Lymphoma
Source: Cancers (Basel). 2020 Sep 1;12(9):2483. doi: 10.3390/cancers12092483 (PMC7563318; doi:10.3390/cancers12092483)
Supplement: Supplementary file 1 [file cancers-12-02483-s001.zip › cancers-871644 supplementary file/cancers-871644-supplementary layout proof read.docx]

Supplementary Materials

Lenalidomide in Combination with Arsenic Trioxide: an Effective Therapy for Primary Effusion Lymphoma

Sara Moodad, Rana El Hajj, Rita Hleihel, Layal Hajjar, Nadim Tawil, Martin Karam,
Maguy Hamie, Raghida Abou Merhi, Marwan El Sabban and Hiba El Hajj


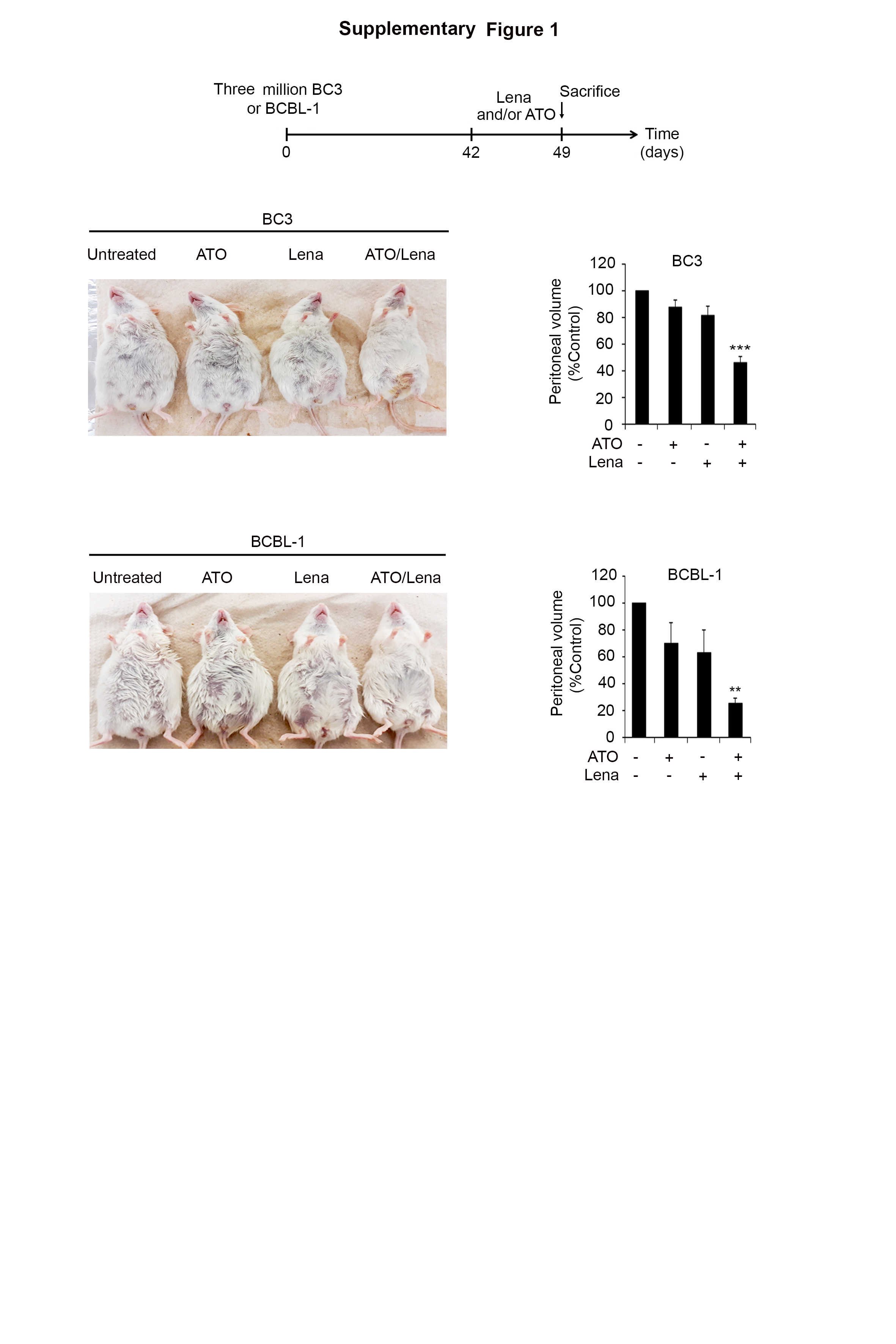


**Figure S1.** ATO/Lena decreased peritoneal volume in PEL mice. Timeline of the treatment with ATO, Lena or their combination. Mice phenotype, and histogram plots of peritoneal volume of BC3 (upper panel) or BCBL-1 (lower panel) mice before and after one week treatment with ATO, Lena or their combination. (**) indicates *p* < 0.01; and (***) indicates *p* < 0.001.


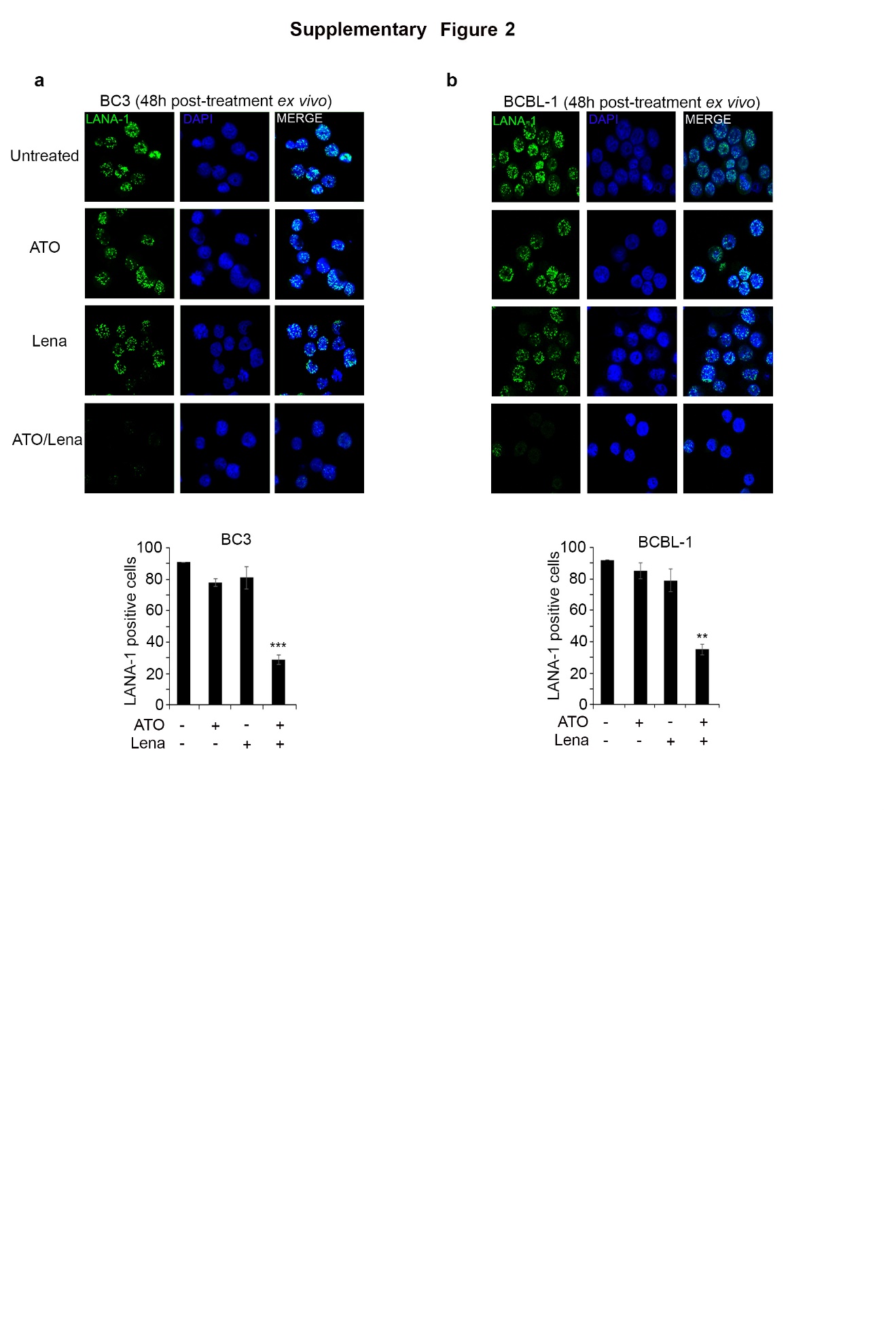


**Figure S2.** ATO/Lena decreased LANA-1 expression in ascites-derived BC-3 and BCBL-1 PEL cells. (**a,b**) Confocal microscopy analysis of LANA-1 expression in ascites-derived BC-3 (**a**) and BCBL-1 (**b**) following ex vivo treatment with ATO/Lena for 48 h. LANA-1 was stained with rat anti-LANA-1 antibody (green). Nuclei were stained with Hoechst (blue). Histograms represent the average percentage of LANA-1 positive cells from three independent experiments. (**) indicates *p* < 0.01; and (***) indicates *p* < 0.001.


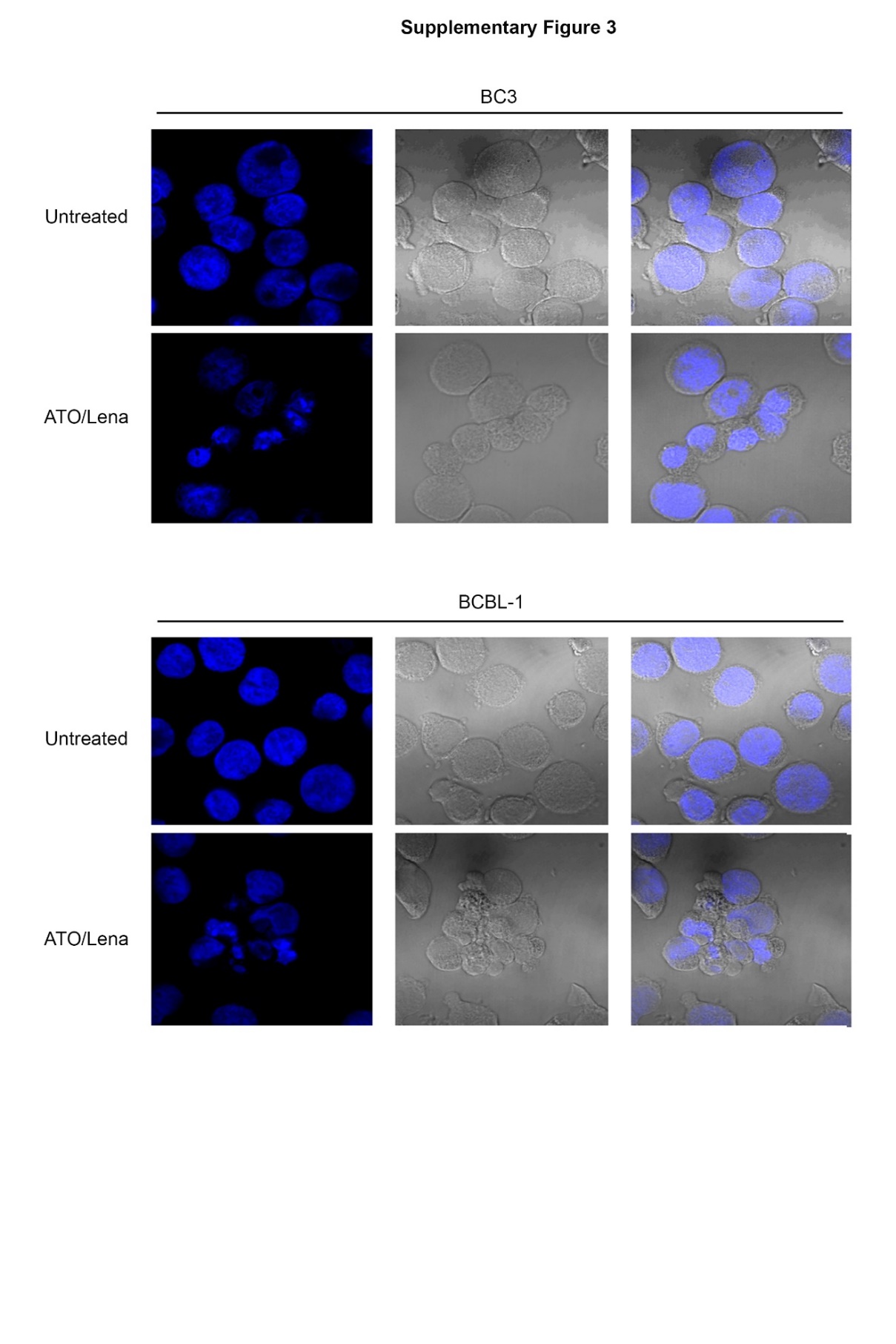


**Figure S3.** ATO/Lena induced apoptosis in ascites-derived BC-3 and BCBL-1 PEL cells. Confocal microscopy analysis of ascites-derived BC3 or BCBL-1 cells after staining with Diamidine-2′-phenylindole dihydrochloride (Dapi). Condensation of chromatin, indicative of apoptosis is obtained 48 h post-treatment with ATO/Lena combination.


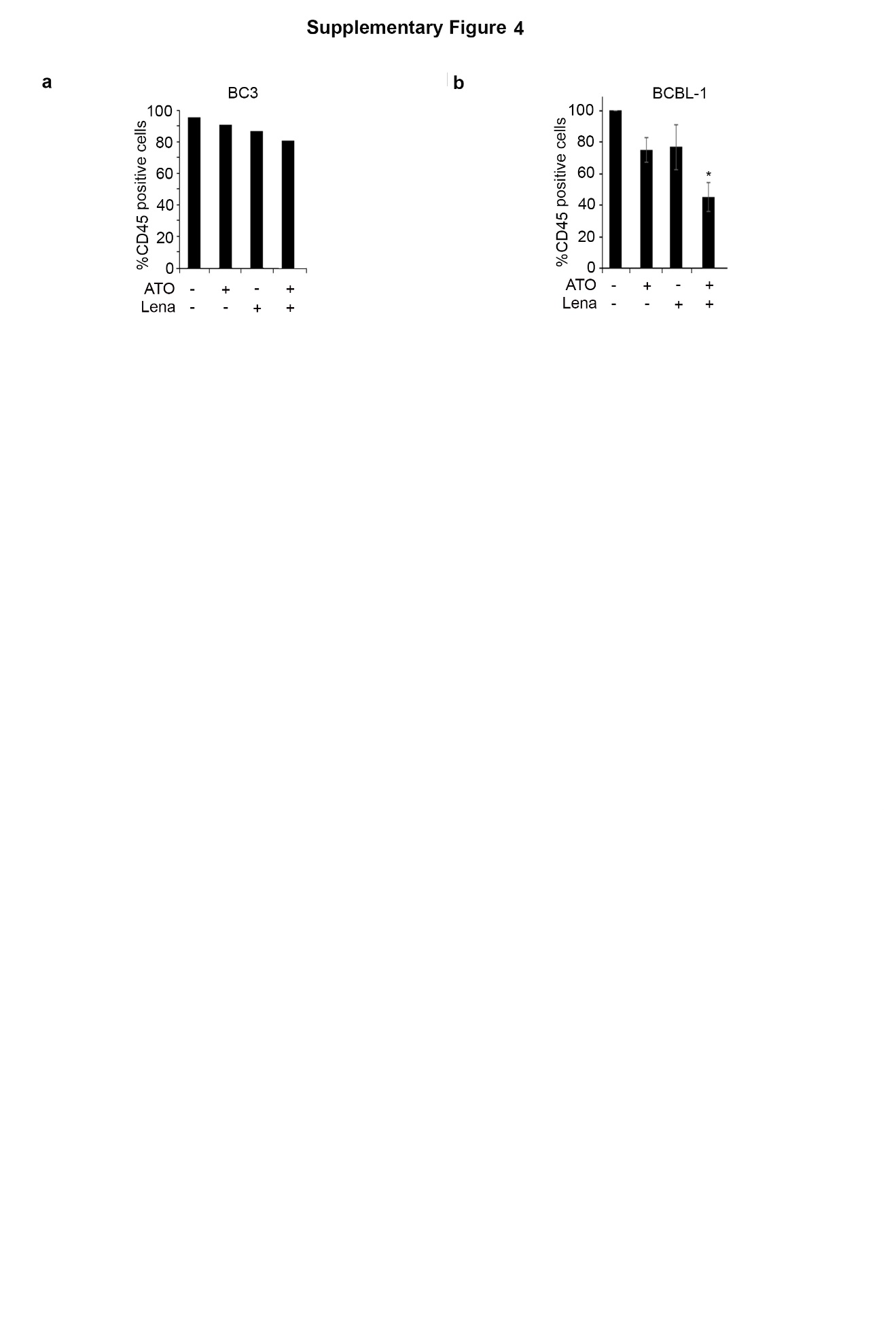


**Figure S4.** CD45 staining of peritoneal ascites from PEL treated mice. (**a,b**) Ascites-derived cells from untreated BC3 and BCBL-1 PEL mice, or treated mice with ATO, Lena single agents or their combination, were stained with human CD45 PE antibody. Histograms represent the percentage of CD45 positive cells in ascites from treated/untreated BC-3 (**a**) or BCBL-1 (**b**) mice.

**Table S1.** List of primers used for RT-qPCR.

| Primer | Sequence 5’-3’ | Reference |
| --- | --- | --- |
| K8.1 Forward Primer | TTCCACACAGATTCGCACAGA | [1] |
| K8.1 Reverse Primer | GGCACGCCACCAGACAA |  |
| ORF59 Forward Primer | CGTCGGTAGCGGCTTCA | [1] |
| ORF59 Reverse Primer | GGCTATGCCAGCGTCGAGTA |  |
| ORFK8 Forward Primer | CAAGAGGCGACTACATAGAAA | [2] |
| ORFK8 Reverse Primer | GATCACATACTTCGGCCTTAAC |  |
| RTA Forward Primer | CGCAATGCGTTACGTTGTTG | [3] |
| RTA Reverse Primer | GCCCGGACTGTTGAATCG |  |
| V-FLIP Forward Primer | GTGTTCATACCTCAACCCACAC | [3] |
| V-FLIP Reverse Primer | CACACAGCTCCCCGTCTAC |  |
| V-Cyclin Forward Primer | TCAGTTTGCCAGGAATACAACCTAG | [3] |
| V-Cyclin Reverse Primer | AAGAAGGAAGTTACGTCCGTCG |  |
| GAPDH Forward Primer | CATGGCCTTCCGTGTTCCTA | [4] |
| GAPDH Reverse Primer | CCTGCTTCACCACCTTCTTGAT |  |
| IL-6 Forward Primer | AACCTGAACCTTCCAAAGATGG | [5] |
| IL-6 Reverse Primer | TCTGGCTTGTTCCTCACTACT |  |
| IL-10 Forward Primer | TCTCCGAGATGCCTTCAGCAGA | [6] |
| IL-10 Reverse Primer | TCAGACAAGGCTTGGCAACCCA |  |

References

1. Di Bartolo, D.L.; Hyjek, E.; Keller, S.; Guasparri, I.; Deng, H.; Sun, R.; Chadburn, A.; Knowles, D.M.; Cesarman, E. Role of defective Oct-2 and OCA-B expression in immunoglobulin production and Kaposi's sarcoma-associated herpesvirus lytic reactivation in primary effusion lymphoma. *J. Virol.* **2009,** *83*, 4308–4315, doi:10.1128/JVI.02196-08
2. Lefort, S.; Flamand, L. Kaposi's sarcoma-associated herpesvirus K-bZIP protein is necessary for lytic viral gene expression, DNA replication, and virion production in primary effusion lymphoma cell lines. *J. Virol.* **2009,** *83*, 5869–5680, doi:10.1128/JVI.01821-08
3. El Hajj, H.; Ali, J.; Ghantous, A.; Hodroj, D.; Daher, A.; Zibara, K.; Journo, C.; Otrock, Z.; Zaatari, G.; Mahieux, R.; et al. Combination of arsenic and interferon-alpha inhibits expression of KSHV latent transcripts and synergistically improves survival of mice with primary effusion lymphomas. *PLoS One* **2013,** *8*, e79474. doi:10.1371/journal.pone.0079474
4. Wisnieski, F.; Calcagno, D.Q.; Leal, M.F.; dos Santos, L.C.; de Oliveira Gigek, C.; Chen, E.S.; Pontes, T.B.; Assumpção, P.P.; de Assumpção, M.B.; Demachki, S.; et al. Reference genes for quantitative RT-PCR data in gastric tissues and cell lines. *World J. Gastroenterol.* **2013,** *19*, 7121–7128, doi:10.3748/wjg.v19.i41.7121
5. Xu, L.; Feng, X.; Tan, W.; Gu, W.; Guo, D.; Zhang, M.; Wang, F. IL-29 enhances Toll-like receptor-mediated IL-6 and IL-8 production by the synovial fibroblasts from rheumatoid arthritis patients. *Arthritis Res. Ther.* **2013,** *15*, R170, doi:10.1186/ar4357
6. Chen, M.; Hu, P.; Ling, N.; Peng, H.; Lei, Y.; Hu, H.; Zhang, D.; Ren, H. Enhanced functions of peripheral γδ T cells in chronic hepatitis B infection during interferon α treatment in vivo and in vitro. *PLoS One* **2015,** *10*, e0120086, doi:10.1371/journal.pone.0120086

| 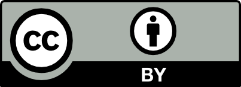 | © 2020 by the authors. Licensee MDPI, Basel, Switzerland. This article is an open access article distributed under the terms and conditions of the Creative Commons Attribution (CC BY) license (http://creativecommons.org/licenses/by/4.0/). |
| --- | --- |
